# Commensal microbiota–coated biohybrid implants induce antibiofilm, osteogenic, and immunomodulatory responses in a human 3D immunocompetent model

**DOI:** 10.1016/j.bioactmat.2026.06.006

**Published:** 2026-06-16

**Authors:** Raunak Lohar, Tayyaba Nawaz, Timm Landes, Paula Schaefer-Dreyer, Philipp-Cornelius Pott, Michael Pflaum, Meike Stiesch, Muhammad Imran Rahim

**Affiliations:** a Department of Prosthetic Dentistry and Biomedical Materials Science, Hannover Medical School, Carl-Neuberg-Str. 1, Hannover, 30625, Germany; b Lower Saxony Centre for Biomedical Engineering, Implant Research and Development (NIFE), Stadtfelddamm 34, Hannover, 30625, Germany; c HOT – Hannover Centre for Optical Technologies, Leibniz University Hannover, Nienburger Str. 17, Hannover, 30167, Germany; d Clinic for Cardiothoracic, Transplantation and Vascular Surgery, Hannover Medical School, Carl-Neuberg-Str. 1, Hannover, 30625, Germany

**Keywords:** Commensal microflora, Biohybrid implants, 3D cell culture model, Biomaterial-associated infections, Osteoimmunomodulation, Immunomodulation, Osteogenesis

## Abstract

Medical devices have revolutionized patient care; however, their abiotic surfaces remain highly susceptible to bacterial biofilm formation, enabling immune evasion and antibiotic resistance. Although antibacterial coatings can mitigate bacterial colonization, many rely on antimicrobial agents that may drive resistance and often lack the capacity to actively modulate host immunity or promote osteogenesis. Here, we introduce innovative Commensal Hybrid Materials (CHMs), multifunctional implant interfaces generated by coating titanium with commensal microflora via a heat-anchoring inactivation process to create a safe, stable surface layer. Surface analyses (SEM/EDS, FTIR, Raman, XPS) confirmed a thick, near-hydrophobic coating with a polar biomolecular overlayer bearing protein (amide), lipid (C–H), and phosphate/phosphoryl signatures. CHMs were non-haemolytic, biocompatible with human cells, enhanced macrophage antimicrobial activity by increasing reactive oxygen species production while maintaining balanced phagocytosis, and reduced biofilm formation by the periodontal pathogen *Porphyromonas gingivalis*. CHMs induced balanced macrophage polarization and osteoimmunomodulation, increasing expressions of *IL-1β*, *TNF-α* (M1), *IL-10* (M2), and the osteoinductive mediator oncostatin M (*OSM*), while significantly upregulating *RUNX2* in co-cultured periodontal ligament stem cells with an early, self-limiting cytokine profile. In a clinically relevant human immunocompetent three-dimensional implant-tissue-oral-bacterial-biofilm (INTER_b_ACT) model integrating fibroblasts, epithelial cells, macrophages, and multispecies biofilms, CHMs preserved epithelial integrity under dysbiotic challenge and significantly reduced biofilm volume while tuning macrophages toward both antimicrobial (M1) and reparative (M2) states. CHMs also reduced pro-inflammatory cytokine expression under both sterile and biofilm-exposed conditions. Collectively, CHMs uniquely combine biofilm resistance, immune-mediated clearance, and osteogenic stimulation, supporting translation to dental and orthopaedic implants.

## Highlights

• Novel biohybrid implants (CHMs) coated with commensal microflora exhibit potent biofilm-repellent activity.
• CHMs mediate osteoimmunomodulatory effects through macrophage–stem cell crosstalk.
• In the human immunocompetent 3D INTER_b_ACT model, CHMs exhibit strong anti-biofilm activity against complex multispecies biofilms.
• CHMs demonstrate biocompatibility along with immunomodulatory and anti-inflammatory activities in 3D tissue models.

## Graphical abstract

**Figure:**
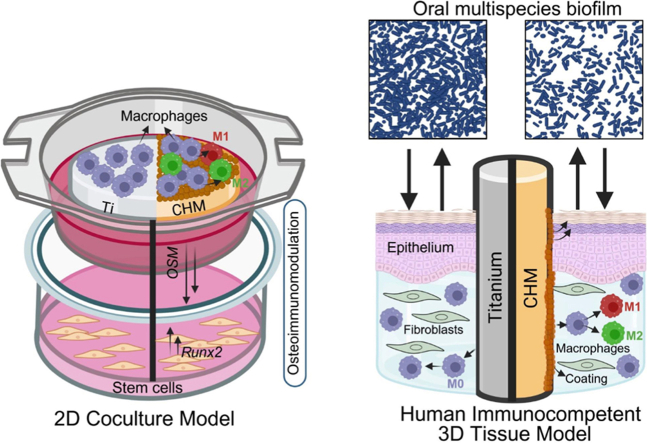

## 1 Introduction

The global use of implantable medical devices to restore or replace diseased or missing body structures continues to rise, driven largely by an aging population and the increasing prevalence of chronic, life-threatening diseases [1]. Despite their clinical benefits, implant surfaces are inherently non-shedding and biologically inert, making them highly vulnerable to bacterial adhesion and subsequent biofilm formation [[2], [3], [4], [5]]. Once established, biofilms on implants provoke persistent inflammation and recurrent infection, often culminating in implant failure and thus representing a major clinical challenge [[6], [7], [8]]. Dental implants are particularly susceptible, as the peri-implant environment fosters the development of complex multispecies biofilms. These biofilms exhibit diverse nutrient requirements, coordinated metabolic activities, and structural resilience, all of which promote their persistence, interspecies synergy, and altered gene expression. Importantly, biofilms display remarkable tolerance to antibiotic therapy, leading to dysbiosis that disrupts local microbial homeostasis and drives progressive peri-implant tissue destruction [9,10].

Various therapeutic strategies, including hydrogel- and nanoparticle-based approaches, are under investigation to control bacterial biofilm infections [[11], [12], [13]]. In the oral environment, commensal, or healthy-associated, microbiota play an essential role in maintaining peri-implant health. Through cooperative interactions with the host immune system, these microorganisms restrict pathogen colonization and preserve tissue homeostasis [[14], [15], [16]]. Acting as natural defenders against dysbiosis, these microorganisms compete with pathogens for adhesion sites, nutrients, and ecological niches [[17], [18], [19]]. At clinically healthy peri-implant sites, the microbial community is typically dominated by *Streptococcus*, *Rothia*, *Neisseria*, and *Corynebacterium* species, together with Gram-positive cocci and facultative anaerobic rods. This consortium maintains microbial balance and prevents pathogenic overgrowth [20]. In peri-implant mucositis, microbial diversity increases, with health-associated taxa coexisting alongside disease-related organisms, including members of the orange and red complexes that contribute to early soft-tissue inflammation. In peri-implantitis, microbial diversity and pathogenic complexity intensify, with the dominance of *Porphyromonas gingivalis*, *Tannerella forsythia*, *Treponema denticola*, and *Fusobacterium nucleatum*, as well as emerging pathogens such as *Filifactor alocis* and *Fretibacterium fastidiosum* [21]. Recent strategies have sought to exploit the protective and immunomodulatory functions of commensal microbiota and probiotics to counteract pathogenic biofilms [22]. Probiotic strains, including *Lactobacillus* and *Bifidobacterium*, have been shown to suppress *Streptococcus mutans* biofilms and inhibit the hyphal growth of *Candida albicans* [[23], [24], [25]]. Moreover, in a human 3D cell culture model, the commensal bacterium *Streptococcus oralis* induced a protective stress response in peri-implant mucosa, reducing IL-6 and IL-8 secretion and thereby attenuating inflammation [26]. Bioengineering approaches that coat implant surfaces with commensal microorganisms further reinforce these benefits, demonstrating enhanced host immune modulation and reduced pathogenic biofilm formation [27,28]. Oral commensal streptococci are regarded as “gatekeepers” of the oral microbiome. As primary colonizers, they help maintain microbial homeostasis by producing adhesins, hydrogen peroxide, bacteriocins, and nitric oxide intermediates that inhibit pathogenic overgrowth [29]. *In vitro, S. oralis* interacts with gingival epithelial cells without causing tissue damage, eliciting only a mild, balanced immune response that supports peri-implant health [30]. It also antagonizes cariogenic species, further contributing to microbial stability [31,32].

Building on this knowledge, we developed commensal hybrid materials (CHMs) by coating implant surfaces with *S. oralis* through a heat-inactivation process that kills the bacteria after anchoring them to the surface, thereby eliminating any risk of sepsis. The rationale for this approach was that *S. oralis*, as a key ecological gatekeeper, could preserve homeostasis and prevent pathogenic colonization. Previous work in our group demonstrated that CHMs effectively inhibited initial bacterial adhesion, the critical first step in biofilm formation [33,34]. In the present study, we systematically evaluated the biocompatibility, biofilm-repellent capacity, and osteo-immunomodulatory properties of CHMs using both a two-dimensional (2D) model comprising macrophages and periodontal ligament stem cells (PDLSCs) and a clinically relevant human three-dimensional (3D) implant–tissue–oral-biofilm (INTER_b_ACT) model with incorporated immune cells [35,36]. Our findings demonstrate that CHMs exert multiple synergistic effects through a single surface modification within this advanced 3D system, highlighting their unique multifunctionality. By simultaneously exhibiting anti-biofilm activities and modulating host immune and tissue responses, CHMs may represent a promising strategy for preventing implant-associated infection and supporting peri-implant homeostasis. Collectively, these results underscore the translational potential of CHMs for *in vivo* validation and future clinical application, where they may provide a novel therapeutic avenue for preventing biomaterial-associated infections and improve the long-term success of implantable biomaterials.

## 2 Material and methods

### 2.1 Synthesis of commensal microflora coated implants

Grade 4 titanium (Ti) discs with machined surfaces were used in two formats: 12 mm in diameter × 2 mm in thickness and 3 mm in diameter × 2.3 mm in height (L. Klein SA, Switzerland). The discs were cleaned in 1% sodium hypochlorite for 30 min in an ultrasonic bath, rinsed in double-distilled water for 20 min, and sterilized by autoclaving prior to cell culture experiments.

*Streptococcus oralis* (ATCC 9811, American Type Culture Collection, Manassas, USA) was cultured for 24 h in Tryptic Soy Broth (TSB) under aerobic conditions at 37°C with constant shaking at 200 rpm. The bacterial suspension was centrifuged and washed twice with double-distilled water to remove traces of culture medium. Optical density at 600 nm was adjusted to 1.5 in double distilled water. This suspension served as the commensal coating solution. Sterile titanium discs were placed on a heating plate at 75°C, and 50 μL of the suspension was applied to each surface. After allowing the bacterial suspension layer to dry, the process was repeated similarly until a total of 20 layers had been deposited to generate the CHM (Fig. S1).

### 2.2 Microstructural and composition analysis of coated surfaces

The surface morphology of Ti and CHM discs was examined by field-emission scanning electron microscopy (FE-SEM) using a Zeiss Merlin microscope (Carl Zeiss, Germany). Elemental composition was assessed by energy-dispersive X-ray spectroscopy (EDS) with an EDAX detector (Mahwah, NJ, USA) coupled to the SEM. Fourier transform infrared (FTIR) spectra were recorded using a Nicolet iS5 spectrometer (Thermo Fisher Scientific) equipped with a triglycine sulfate (TGS) detector and an attenuated total reflection (ATR) accessory with a pressure arm and diamond/ZnSe crystal. Spectra were collected in the range 4000–525 cm^−1^ at 4 cm^−1^ resolution, averaging 16 co-added scans per measurement. An uncoated titanium disc was measured under identical conditions and used as the background reference prior to acquisition of spectra from coated samples. Ti and CHMs were investigated by a custom-built confocal Raman microscope. 532 nm laser light (Cobolt 04-01 Samba, Hübner Photonics/Cobolt AB, Solna, Sweden) was coupled to a custom automated stage-scanning microscope. The light was focused using a long working distance 20x objective (M Plan Apo; Mitutoyo, Takatsu-ku, Japan) with a numerical aperture (NA) of 0.42. The scattered light was collected with the same objective in a backscattering geometry. A dichroic mirror (Di03-R532-t3-25x36, IDEX Semrock, Rochester, New York, USA) and a subsequent notch filter (NF533-17, Thorlabs, Newton, New Jersey, USA) filtered the excitation light, while transmitting the Stokes part of the Raman-spectrum. The light was spatially filtered using an optical fiber acting as a pinhole and spectrally analysed with a Czerny-Turner spectrometer (iHR320, Horiba Jobin Yvon Ltd., Kyoto, Japan) connected to a deep-cooled CCD-senor (Syncerity BI UV-VIS, Horiba Jobin Yvon Ltd., Kyoto, Japan). Overview spectra were acquired using a 600 lp/mm grating, 2 s integration time and 3 mW of optical power, while detailed scans of the fingerprint region were acquired with a 1800 lp mm grating, 5 s integration time, and 10 mW, respectively. Fingerprint region was sampled on 10 random positions on the sample with 10 accumulations, while overview scans were performed on a region 50 × 50 μm2 with two accumulations. Raman spectra were analysed using RamanSPy [37]. In short, cosmic rays were removed, the spectra were then smoothed using a Savitzky–Golay algorithm (window size of 21 and first-degree polynomial order), baseline-corrected, and normalized. The presence of the coating was verified with band assignment to the CH-NH region (2850–3100 cm-1) and the phenylalanine stretch in the fingerprint region (1000 cm-1) [38]. XPS measurements were performed using a PHI VersaProbe III system (Physical Electronics GmbH). The chemical states of the materials were analysed using microfocused Al Kα radiation (hν = 1486.7 eV; 50 W; 15 kV) with an X-ray spot diameter of 200 μm. During acquisition, dual-beam charge neutralization was applied using a 1 V electron beam and a 7 V Ar^+^ ion beam. Data were processed and evaluated with MultiPak software (ULVAC-PHI). Peak fitting was performed by deconvoluting the C 1s signal into four components and the O 1s and N 1s signals into two components each, corresponding to the assigned chemical states [39,40].

The surface roughness of Ti and CHMs was measured using an optical profilometer (MicroProf 100, FRT GmbH, Bergisch Gladbach, Germany). Surface wettability of the CHMs was evaluated by static water contact angle measurements. Briefly, a 3 μL droplet of ultrapure water was carefully dispensed onto the surface of Ti and CHM discs. Droplet images were acquired using an OCA40 contact angle measurement system (DataPhysics Instruments GmbH, Germany). For each image, the baseline was defined and the left and right contact angles were determined using (OCA 40, DataPhysics Instruments GmbH, Filderstadt, Germany) software. The reported water contact angle was calculated from these measurements.

CHMs were first sterilized by exposure to ultraviolet light for 15 min and then incubated in liquid tryptic soy broth (TSB) for 24 h at 37°C under aerobic conditions. The resulting supernatants were collected, and optical density at 600 nm (OD_600nm_) was measured using fresh TSB as a blank. To further assess microbial contamination, 100 μL aliquots of each supernatant were plated onto FAA-blood agar using the spread-plate method and incubated aerobically at 37°C for 7 days. Plates were then imaged with a digital camera (Nikon D7100, Nikon Corp., Japan) to examine bacterial colony formation. Sterile Ti discs served as the control.

Sterilized CHM discs were washed twice with PBS and stained with 0.1% crystal violet for 20 min at room temperature to visualize the inactivated commensal coating. After removal of excess stain, the discs were washed three times with ultrapure water and air-dried completely. Images of the stained surfaces were captured before the bound crystal violet was solubilized with 30% acetic acid for 15 min. The solubilized dye was then transferred to a 96-well plate, and absorbance was quantified at 590 nm. Sterile Ti discs were included as controls.

The mechanical integrity of the CHMs was first evaluated using a dry tape test. Briefly, 3M 600 tape (Scotch®^,^ USA) was applied to the coated surface, pressed uniformly for 10 s using a rubber roller, and removed after 90 s. The discs were subsequently stained with SYTO 9 and propidium iodide (PI) and imaged by Confocal Laser Scanning Microscope (CLSM; Leica TCS SP8, Leica Microsystems, Mannheim, Germany) at 2.5× magnification to visualize a larger surface area. To assess coating stability under physiological conditions, CHM discs were incubated in Dulbecco's Modified Eagle Medium (DMEM) supplemented with 10% fetal bovine serum (FBS) and 1% penicillin/streptomycin (P/S) for 24 or 72 h under either static (no shaking) or dynamic (continuous shaking at 100 rpm) conditions at 37°C, 5% CO_2_, and 95% relative humidity. Following incubation, the discs were stained and imaged as described above. Images obtained after the dry and wet stability tests were compared with those of freshly prepared CHM discs. Coating retention was quantified by volumetric image analysis to account for potential non-uniform loss of the commensal coating.

Sterile Ti and CHM discs were incubated with fetal bovine serum (FBS) and plasma derived from donated human blood for 24 h at 37°C under 5% CO_2_ and 95% relative humidity to enable protein adsorption onto the material surface. Following incubation, the supernatants were collected and stored at −80°C. The discs were then gently washed twice and subsequently incubated in PBS to facilitate the release of adsorbed proteins. After an additional 24 h, the resulting supernatants were collected and stored at −80°C. Total protein concentrations in all supernatants were quantified using the Pierce™ Bradford Plus Protein Assay Kit (Thermo Scientific, USA).

### 2.3 Cultivation of human cell lines on implant surfaces

Human gingival fibroblasts (HGFs; 121 0412, Provitro GmbH) were cultured in Dulbecco's Modified Eagle Medium (DMEM, PAN Biotech) supplemented with 10% fetal bovine serum (FBS; P30-3309, PAN-Biotech GmbH) and 1% penicillin–streptomycin (P/S, 10,000 U/mL, A2212, Biochrom AG). Human monocytes (THP-1) were cultured in RPMI 1640 (PAN Biotech) with 10% FBS and 1% P/S and differentiated into macrophages using 25 ng/mL phorbol 12-myristate 13-acetate (PMA; Sigma-Aldrich). Human oral keratinocytes (OKF6/TERT-2) were maintained in Keratinocyte-SFM basal medium (Gibco) supplemented with 0.3 mM CaCl_2_, 0.2 ng/mL EGF, 25 μg/L bovine pituitary extract (BPE), and 1% P/S. All cells were incubated at 37°C, 5% CO_2_, and 95% humidity.

HGFs, keratinocytes, and macrophages were individually seeded (1 × 10^5^ cells/sample) onto 12 mm glass, Ti, or CHM discs placed in 24-well plates and allowed to adhere for 30 min. Appropriate growth media were added, and the cultures incubated for 48 h. Cells were stained with 1:1000 Calcein AM (C3099, Thermo Fisher Scientific Inc., Waltham, USA) and 1:1000 propidium iodide (P4864, Sigma-Aldrich Corporation, St. Louis, USA) for 30 min, washed with phosphate-buffered saline (PBS), and imaged using a CLSM at 400× magnification. Calcein AM and PI were excited at 488 nm and 552 nm and detected at 500–550 nm and 650–750 nm, respectively.

### 2.4 Assessment of cell viability, hemolysis, reactive oxygen species production, phagocytosis and immunostaining

HGFs, keratinocytes, and macrophages were seeded at 1 × 10^5^ cells/sample onto glass, Ti, or CHMs and incubated under standard conditions. After 48 h, CellTiter-Blue (Promega, Mannheim, Germany) reagent was added to the medium (1:5 ratio), incubated for 4 h, and fluorescence was measured using a spectrophotometer (Tecan Infinite 200 Pro, Tecan Austria GmbH) at 560 nm with a 590 nm reference wavelength.

To evaluate the potential release of toxic or inflammatory metabolites from the coated surfaces under physiological conditions, CHMs were incubated in RPMI cell culture medium for 24 h. Macrophages were seeded in 6-well plates at a density of 1 × 10^5^ cells/well and cultured for 24 h before exposure to the conditioned medium collected following CHM incubation. After 24 h of incubation, the cells were fixed with 4% paraformaldehyde, permeabilized, and stained with DAPI (Sigma-Aldrich, Taufkirchen, Germany) and Phalloidin-TRITC (Phalloidin-iFluor Reagent; Abcam, Cambridge, UK). Changes in cell morphology were subsequently assessed using CLSM.

Hemolysis was assessed in close accordance with ISO 10993-4 and ISO 10993-12 guidelines for the biological evaluation of blood-contacting medical devices. CHM discs were placed in 12-well plates and embedded in medical-grade silicone (rema® Sil, Dentaurum, Germany) such that only the coated disc surface was exposed to blood. Human blood was obtained from a healthy volunteer in lithium heparin tubes (Sarstedt, Germany) after informed consent and in accordance with institutional ethical approval. Hemoglobin concentration was measured using an ABL90 analyzer (Radiometer, Germany). CHMs and Ti discs were incubated with 400 μL whole blood for 1 h at 37°C under orbital shaking. After incubation, blood samples were collected and centrifuged at 500 × g for 5 min. An aliquot of 100 μL supernatant was transferred to a 96-well plate, and absorbance was recorded at 540 nm. Baseline hemoglobin values were determined using a 1:10 dilution of fresh blood plasma, whereas the positive hemolysis control was generated by 1:10 dilution of whole blood in red blood cell lysis solution (Miltenyi, Germany). The percentage of hemolysis was calculated and compared between Ti and CHM groups.

For reactive oxygen species (ROS) staining, macrophages (1 × 10^5^ cells/mL) were cultured on Ti and CHMs for 48 h and subsequently stained with the DCFDA/H_2_DCFDA Cellular ROS Assay Kit (ab113851; Abcam, Cambridge, UK) according to the manufacturer's instructions. Fluorescence signals were acquired byCLSM, and images were processed using Imaris software. Macrophage phagocytosis was assessed after culture on Ti or CHM surfaces using a phagocytosis kit (ab234053; Abcam, Cambridge, UK). Macrophages (1 × 10^5^ cells/mL) were seeded onto the respective substrates and incubated for 48 h. The kit protocol was followed for subsequent steps. The phagocytotic control group comprised cells pretreated with cytochalasin D (20 μM) for 1 h at 37°C to inhibit actin-dependent internalization. Fluorescent zymosan particles (ab234053; Abcam, Cambridge, UK) were then added in a volume of 5 μL per sample to the cultured cells and allowed to interact for 2 h at 37°C. Following incubation, non-internalized particles were quenched using 0.2% trypan blue. The cells were then stained with DAPI and Phalloidin-TRITC for nuclear and f-actin staining respectively, and imaged CLSM. Image analysis and quantification of internalized particles were performed using Imaris software and ImageJ.

For macrophage immunostaining, THP-1-derived macrophages (1 × 10^5^) were cultured on sterile Ti and CHM discs. After 24 h, cells were fixed with 4% paraformaldehyde, washed with Tris-buffered saline (TBS), and blocked with 3% goat serum containing 0.1% Tween 20 for 30 min. Primary antibodies - (rabbit polyclonal anti-CD80, Invitrogen) for M1 and (mouse monoclonal anti- CD206, clone 2A6A10, Proteintech) for M2-were then applied at the appropriate dilution and incubated overnight at 4°C with gentle shaking. The following day, the antibody solution was removed, and the samples were washed prior to incubation with secondary antibodies - Alexa Fluor™ 488-conjugated goat anti-rabbit IgG (H + L) (Invitrogen) and Cy™5-conjugated AffiniPure™ donkey anti-mouse IgG (H + L) (Jackson ImmunoResearch Laboratories Inc., USA) for 2.5 h. Samples were subsequently washed, counterstained with DAPI, and imaged by CLSM. Samples incubated with secondary antibodies only, in the absence of primary antibodies, served as no-primary controls to check for nonspecific binding of the secondary antibodies.

### 2.5 Bacterial-repellent properties and assessment of surface protein adsorption of commensal microflora-coated implants

*Porphyromonas gingivalis* (DSM 20709, DSMZ, Braunschweig, Germany) with OD_600_ adjusted to 0.1 was cultured on 12 mm glass, Ti and CHM discs in brain heart infusion (BHI) broth and allowed to form a biofilm under anaerobic conditions for 24 h. After 24 h, samples were stained with Syto-9 (Invitrogen, Germany) and PI and live/dead imaged using the CLSM. For simultaneous seeding of bacteria and cells, macrophages were seeded (1 × 10^5^ cells/sample) on 12 mm glass, titanium and CHMs for 30 min. Planktonic *P. gingivalis* was added (10 μL, OD_600nm_ = 0.1) to disc surfaces, with or without macrophages, and incubated for 30 min before macrophage medium (RPMI + 10% FBS) was added. After 24 h, samples were stained with Calcein AM and PI. Culture supernatants were streaked on FAA agar with 5% sheep blood and incubated anaerobically for up to 5 days. Colonies were counted upon appearance. Syto9 and propidium iodide from the LIVE/DEAD® BacLight™ Bacterial Viability Kit (Fisher Scientific GmbH) were diluted 1:1000 in PBS and applied for 30 min on discs. The coated samples were analysed using CLSM.

Sterile Ti and CHM discs were incubated for 24 h with either fetal bovine serum (FBS) or human plasma obtained from donated blood at 37°C under 5% CO_2_ and 95% relative humidity to allow protein adsorption onto the material surface. After incubation, the supernatants were collected and stored at −80°C. The discs were subsequently washed twice gently and incubated in PBS to release surface-adsorbed proteins. After 24 h, the PBS fractions were collected and stored at −80°C. Total protein concentrations in all supernatants were determined using the Pierce™ Bradford Plus Protein Assay Kit (Thermo Scientific, USA).

### 2.6 Gene expression, cytokine profile, ALP activity and mineralization analyses in a co-culture of macrophages on commensal microflora-coated implants with PDLSCs

PDLSCs were seeded (1 × 10^5^ cells/well, α-MEM + 10% FBS + 1% P/S) in 6-well plates. THP-1–derived macrophages (1 × 10^5^ cells/sample) were seeded on 12 mm glass, Ti and CHM discs in transwell inserts for 30 min and then transferred into PDLSCs-containing wells. Inserts were covered with macrophage medium and co-cultured for 3, 7 and 14 days. Total RNA was extracted (Nucleospin RNA kit, Bioanalysis) from both PDLSCs and macrophages and RNA concentration was measured using the nanodrop (Thermo Scientific Nanodrop 2000c). qRT-PCR (LightCycler® 96, Roth) was used to quantify *IL-1β, TNF-α, IL-8, IL-10,* and *OSM* gene expression in macrophages and *ALPL, COL1a1, Runx2*, and *BGLAP* expression in PDLSCs. C_T_ values were obtained using the LightCycler® 96 software, and the 2^−ΔΔCT^ method was used to calculate relative mRNA expression fold change. For multiple cytokine analysis, 500 μL aliquots of culture supernatants were collected from the 2D model, centrifuged to remove debris, and stored at −80°C.

Mineralization in PDLSCs were assessed in the above setup using Alizarin red staining. After 3 days, when PDLSCs had reached confluence, α-MEM was replaced with osteogenic medium containing 10% FBS, 1% P/S, 500 μg/mL ascorbic acid, 1 M β-glycerophosphate, and 10 μM dexamethasone. Medium was changed every 2 days. After 14 days of total culture, inserts were removed and PDLSCs were washed with PBS, fixed in 4% paraformaldehyde for 20 min, rinsed with distilled water, and stained with 2% Alizarin Red S solution (pH 4.2) for 30 min. After washing, images were acquired using a Leica DMi1 inverted microscope (Leica Microsystems CMS GmbH, Germany). For quantification, bound stain was extracted in 10% acetic acid/20% methanol for 30 min at room temperature, and absorbance was measured at 405 nm using an Infinite 200 PRO microplate reader (Tecan, Germany).

### 2.7 Assembly of the 3D peri-implant mucosa model

The INTER_b_ACT model was generated as described previously [35]. Briefly, HGFs were seeded (9 × 10^5^ cells/implant) onto Ti or CHM cylinders and incubated for 96 h. Collagen gel bases (PureCol®, Advanced BioMatrix) prepared in 6-well transwell inserts were seeded with HGFs and THP-1 macrophages (4 × 10^5^ each) and cultured for 72 h. The cell-seeded gel constructs were released, contracted, and punched centrally to insert fibroblast-colonized Ti and CHM cylinders. OKF6 keratinocytes (1 × 10^6^) were added to form the epithelial layer (Fig. S9). Following 14 days of air–liquid interface culture, the tissues were co-cultured for 24 h with multispecies oral biofilms, comprising *Streptococcus oralis* (ATCC® 9811™, American Type Culture Collection ATCC, Manassas, VA, USA), *Actinomyces naeslundii* (DSM 43013), *Veillonella dispar* (DSM 20735), and *Porphyromonas gingivalis* (DSM 20709), German Collection of Microorganisms and Cell Cultures (DSMZ, Braunschweig, Germany). Bacterial stocks were stored at −80°C and routinely cultivated in brain heart infusion medium (BHI; Oxoid, Wesel, Germany) supplemented with 10 μg/mL vitamin K (Roth, Karlsruhe, Germany) under anaerobic conditions (80% N_2_, 10% H_2_, 10% CO_2_) at 37°C prior to experimentation [41]. Controls included sterile glass slips. Tissues were harvested for histology, CLSM imaging, RNA extraction, and flow cytometry.

### 2.8 Histology of 3D peri-implant tissues

Implant-integrated tissues were fixed for 24 h in 4% paraformaldehyde and embedded in Technovit®. Laser sectioning of the tissues and Elastica van Gieson staining of the sectioned tissues was performed by LLS Rowiak LaserLabSolutions GmbH (Hannover, Germany). The sections were imaged using the Zeiss Axioscope 40 microscope combined with a Zeiss AxioCam Mrc digital camera.

### 2.9 Imaging of live/dead stained tissue and multi-species biofilm

Ti- and CHM-integrated tissues, after coculture under sterile or biofilm-infected conditions, were longitudinally sectioned at the tissue–implant interface and stained with Calcein AM and propidium iodide (Invitrogen, Germany) for 30 min. The stained tissues were washed once with PBS and then imaged using the CLSM. Calcein AM and propidium iodide were excited using 488 nm and 552 nm laser lines and were detected from 500 to 550 nm and 650–750 nm, respectively. The oral multispecies biofilm on glass cover slips after co-cultivation with Ti- or CHM-integrated tissue were washed twice with PBS and stained with Syto-9 and Propidium iodide for 30 min. The staining solution was washed away once with PBS and the biofilms imaged using the CLSM. The Ti and CHM implants were removed from the tissues after co-culture under sterile or biofilm-infected conditions. The implants were stained with Calcein AM and Propidium iodide for 30 min, washed once with PBS and then imaged using the CLSM to examine surface cellular adhesion and coating stability after the peri-implant model development.

### 2.10 Relative mRNA expression quantification

After implant removal, the residual soft tissues were used to extract total RNA using the Nucleospin® RNA isolation kit (Bioanalysis). RNA concentration was measured using the nanodrop (Thermo Scientific Nanodrop 2000c). cDNA was synthesized using the QuantiTect Reverse Transcription Kit (Qiagen) and qRT-PCR was performed using the LightCycler® 96 (Roth). The LightCycler® 96 software was used to obtain the C_T_ values, and the 2^−ΔΔCT^ method was used to calculate relative mRNA expression fold change. mRNA expressions were measured for the following human genes: *IL-1β, TNF-α, IL-8, IL-10, OSM*, (Taqman®).

### 2.11 Flow cytometry analyses and cytokine expression profiling

Ti- and CHM-integrated tissues under sterile and biofilm-cocultivated conditions were subjected to dissociation using Collagenase D (Roche Diagnostics, GmbH) for 45 min to obtain resident cell populations. The cells were counted and distributed equally (2.5 × 10^5^ cells) across three groups ‒ unstained panel (control), antibody panel 1 (CD163, CD86, CD326 and CD14) and antibody panel 2 (CD90, CD45 and CD206). REAfinity anti-human antibodies were obtained from Miltenyi Biotec. Cells in panel 1 and 2 were first stained using Zombie Aqua fixable viability dye (Biolegend) and then stained with the respective antibody panels. Flow cytometry was performed using the MACSQuant® Analyzer 10 flow cytometer (Miltenyi Biotec) with a 25,000-event count limit from each sample. The FlowJo™ 10 (Version 10.10.0) software was used to analyse the flow cytometry data and generate statistical values. An unstained mix of hGF, OKF6, THP-1 monocytes and macrophages (M0 differentiated as well as M1 and M2 polarized) was used as the gating control. The data was analysed using the following gating strategy: (1) The cell population was gated from possible debris using the SSC (side scatter) vs FSC (forward scatter) plot. (2) The singlet cells were gated and separated from the doublets or multiples using the SSC-height vs SSC-area plot. (3) The zombie aqua negative cells were selected by gating to ensure that data was analysed from only living cells. (4) For each fluorochrome channel in the respective panels, a baseline for fluorescence intensity was set higher than the obtained events to ensure the elimination of autofluorescence noise. (5) Keeping this baseline, positive antibody-conjugated fluorochrome event count for each sample was calculated as a percentage of the total events. For multiple cytokine analysis, culture supernatants (500 μL) were collected from the 2D and 3D models, clarified by centrifugation, and stored at −80°C. The concentrations of GM-CSF, IFN-γ, IL-2, IL-4, IL-6, IL-8, IL-10, and TNF-α were quantified using the Bio-Plex Pro Human Cytokine 8-plex Assay (#M50000007A; Bio-Rad, Germany) according to the manufacturer's instructions. IL-1β expression levels were measured using an enzyme-linked immunosorbent assay (ELISA) using the Invitrogen Human IL-1beta ELISA kit (Bender MedSystems GmbH, Austria)

### 2.12 Graphical representation and statistical analysis

GraphPad Prism 10 (Version 10.4.1) was used to graphically represent experimental data and perform statical analyses. Analyses between effects from material groups at a single time point were performed using One-way ANOVA, while grouped data was analysed using Two-way ANOVA. Statistical significance was set using α = 0.05. CLSM live-dead image analyses as well as biofilm volume quantification was performed using the Imaris software. All other images were analysed using ImageJ software (Version 2.1.0/1.53c). Image illustrations were performed using Biorender, ImageJ and Inkscape (Version 1.4).

## 3 Results

### 3.1 Characterization of commensal microflora coatings on titanium implant surfaces

**Fig. 1:**
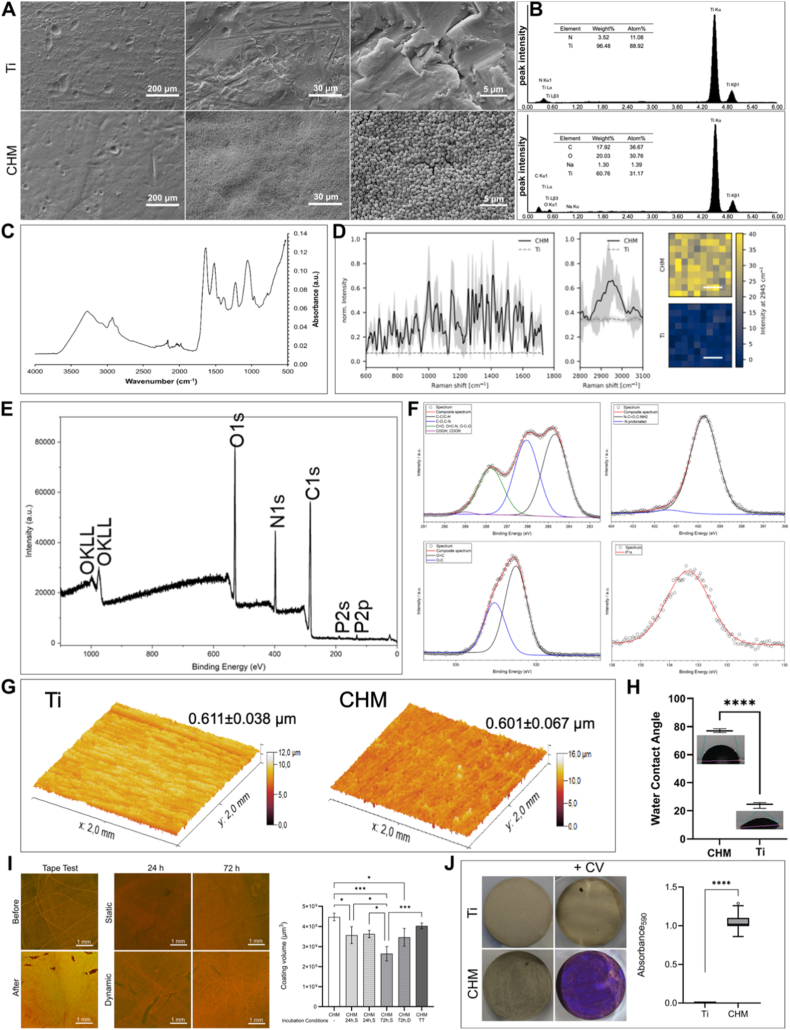
Structural and physicochemical characterization of commensal microflora coatings. (A) SEM images of Ti and CHM discs, shown at increasing magnifications (left to right). (B) EDS of Ti (top) and CHM (bottom) surfaces, confirming titanium as the main element in uncoated samples and detectable levels of carbon, oxygen, and sodium on coated surfaces. Scale bars (left to right): 200 μm, 30 μm, and 5 μm. (C) FTIR analysis of CHM surface, (D) Raman spectrophotometric analysis of CHM and Ti, (E) XPS analysis of CHM, High-resolution scans for carbon (F), nitrogen, oxygen and phosphorus. Surface roughness of Ti and CHM surface (G). Water contact angle (H), CLSM images illustrating coating biomass and quantitative coating volume on Ti and CHM surfaces after 24 and 72 h under static and dynamic conditions and after the tape test (I), Crystal violet staining of Ti and CHM surfaces with corresponding OD600 measurements (J). All analyses were performed with n = 6. For coating stability analysis, samples were examined by CLSM at 2.5× magnification. The voxel size was 4.53 μm × 4.53 μm × 20 μm in the x, y, and z dimensions, respectively.

SEM analysis of the coated implant surfaces revealed the formation of a thick, uniformly distributed layer across the entire surface (Fig. 1A–CHM). At higher magnification, commensal microflora were clearly visible adhering to the implant surface. Energy-dispersive X-ray spectroscopy of Ti surfaces confirmed titanium as the predominant element (Fig. 1B–Ti). In contrast, coated surfaces primarily consisted of carbon (C), oxygen (O), sodium (Na), and titanium (Fig. 1B and CHM). The FTIR spectrum of the coated surface was consistent with previous FTIR findings and with the classification [42]. Compared with Ti, CHMs showed additional absorption bands corresponding to O–H and N–H stretching modes (4000–3100 cm^−1^), C–H stretching vibrations of –CH_3_ and >CH_2_ groups associated with fatty-acid chains of membrane amphiphiles (3100–2800 cm^−1^), amide I and II bands arising from protein amide groups (1800–1500 cm^−1^), and bending modes of >CH_2_ and >CH_3_ from lipids and proteins (1500–1300 cm^−1^) (Fig. 1C). Additional bands included ∼1230 cm^−1^ (P=O asymmetric stretching, attributed to phospholipids), 1200–900 cm^−1^ (PO_2_^−^ symmetric stretching, attributed to phosphate-containing biomolecules including nucleic acids), and signals in the 900–600 cm^−1^ fingerprint region (Fig. 1C). Raman spectra were collected in three modes: fingerprint, high-wavenumber C–H/N–H, and spatial mapping of coated vs uncoated regions on a Ti substrate (Fig. 1D). The fingerprint region showed multiple defined bands consistent with an organic/proteinaceous signal superimposed on Ti-related contributions. In the fingerprint region, key protein and aromatic markers were observed: Amide I (∼1650 cm^−1^) and Amide III (∼1230–1270 cm^−1^) indicative of peptide backbone vibrations and secondary-structure sensitivity, alongside aromatic residue bands at ∼1000 cm^−1^ (phenylalanine), ∼1350 cm^−1^ (tryptophan Fermi resonance), and ∼850 cm^−1^ (tyrosine Fermi resonance). Some weaker features may overlap with Ti-derived resonances/background. The high-wavenumber region exhibited a broad, overlapped C–H stretching envelope with contributions from aromatic C–H (∼3061 cm^−1^), CH_2_ stretches (∼2850–2900 cm^−1^), CH_3_ stretches (∼2875–2920 cm^−1^), and methine CH (∼2971 cm^−1^), consistent with mixed organic content. Spatial Raman scans clearly distinguished coated from uncoated areas: coated regions showed enhanced Amide and aromatic bands and a stronger C–H stretching envelope, whereas uncoated areas were dominated by the Ti substrate response (Fig. 1D).

X-ray photoelectron spectroscopy (XPS) spectrum showed prominent signals corresponding to C 1s (∼285 eV), N 1s (∼399–401 eV), O 1s (∼530–533 eV), and P 2p/P 2s (∼133/∼190 eV), consistent with a bio-derived overlayer on the titanium surface (Fig. 1E). High-resolution scans verified the presence of carbon, nitrogen, oxygen, and phosphorus in the outermost surface region, indicating enrichment of organic and phosphate-containing chemical functionalities following coating. The C 1s signal suggested a carbon-rich surface layer typical of biological material, while the presence of N 1s indicated nitrogen-containing functional groups (e.g., amines/amides) associated with bacterial cell-envelope components and proteinaceous material (Fig. 1F). The strong O 1s contribution reflected oxygenated functionalities and may include contributions from the underlying titanium oxygen-containing groups within the bacterial coating (Fig. 1F). Importantly, the detection of phosphorus (P 2p/P 2s) supported the presence of phosphate/phosphoryl-containing moieties, which are consistent with bacterial membrane and cell-wall constituents (Fig. 1F).

The surface roughness of the commensal microflora–coated implant was 0.601 ± 0.067 (Ra) (Fig. 1G). The water contact angle of the coated surface was close to 90°, indicating a predominantly hydrophobic surface character (Fig. 1H). The bioburden assay indicated that coated bacteria were non-viable and no growth was observed on agar plates (Fig. S2). Supernatants collected from CHMs after 24 h of incubation in cell culture medium were non-toxic and supported macrophage growth and morphology comparably to cells cultured with medium collected from titanium surfaces used as controls (Fig. S3). Tape testing demonstrated strong coating adhesion, with the majority of the commensal microflora layer remaining visibly intact on the implant surface after tape removal (Fig. 1I). Coating integrity was further maintained following incubation in cell culture medium for 24 and 72 h under both static and dynamic conditions (Fig. 1I). Consistent with these observations, quantitative coating-volume measurements showed no appreciable loss of coating mass after the tape test or after 24 and 72 h of incubation in medium under either condition, indicating robust coating stability on the implant surface (Fig. 1I). CHMs surfaces showed strong crystal violet staining, whereas uncoated titanium showed little to no crystal violet staining on its surface (Fig. 1J). Taken together, these findings confirm that a stable, multilayered, and hydrophobic organic coating, enriched in protein as well as nitrogen- and phosphorus-containing biochemical groups characteristic of bacterial biomass, can be successfully established on titanium implants.

### 3.2 Biocompatibility of commensal microflora-coated implant surfaces with tissue-specific human cells

**Fig. 2:**
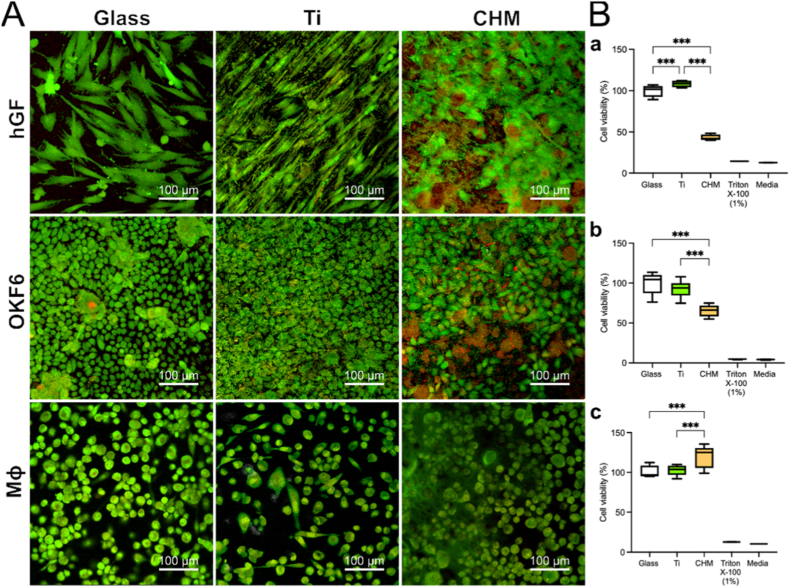
Commensal microflora coatings support adhesion and viability of human cells. (A) CLSM images showing the morphology and distribution of gingival fibroblasts, oral keratinocytes, and macrophages after 48 h on glass, Ti, and CHM. Scale bar: 100 μm. (B) Metabolic activity of (a) fibroblasts, (b) keratinocytes, and (c) macrophages after 48 h on the indicated surfaces. Cells were stained with green fluorescent Calcein AM to label viable cells and with red fluorescent propidium iodide (PI) to label nonviable (dead) cells. All experiments were conducted using three biological replicates (N = 3) and three technical replicates per group (n = 3). For CLSM, the biocompatibility assay was imaged at 10× magnification with a voxel size of 1.52 μm (x) × 1.52 μm (y) × 3 μm (z).

The biocompatibility of coated implant surfaces was assessed using tissue-specific human cell lines commonly found in peri-implant regions. Human gingival fibroblasts, oral keratinocytes, and THP-1 monocyte-derived macrophages were seeded onto Ti and CHM surfaces and cultured for 48 h. Following incubation, cells were stained and imaged using CLSM. CHM surfaces supported the adhesion and growth of human gingival fibroblasts, comparable to titanium and glass controls (Fig. 2A). Similarly, epithelial cells exhibited good adhesion and morphology on coated surfaces, consistent with those observed on control surfaces (Fig. 2A). Macrophages also showed favorable compatibility with the commensal microflora coating, displaying morphology similar to Ti controls (Fig. 2A). To further assess cell viability, metabolic activity was quantified using a metabolic assay. Human gingival fibroblasts demonstrated high metabolic activity on Ti and glass, but reduced activity on coated surfaces (Fig. 2B–a). Epithelial cells showed moderately decreased metabolic activity on coated surfaces compared to controls (Fig. 2B–b). Interestingly, macrophages exhibited significantly enhanced metabolic activity and viability on commensal microflora-coated surfaces compared to controls (Fig. 2B and c). Overall, these findings demonstrate that commensal microflora coatings effectively support the adhesion of tissue-specific human cells while enhancing macrophage viability, thereby maintaining biocompatibility and promoting immune cell functionality. A haemolysis assay demonstrated low haemolytic activity for both CHM and Ti. The CHMs showed haemolysis of up to 2.5%, compared with 1.4% for Ti, indicating minimal erythrocyte lysis and overall hemocompatibility (Fig. S4A and B). Bradford analysis of post-incubation supernatants showed no significant difference in remaining protein concentration between Ti and CHM in either plasma or FBS (Fig. S4C). This indicated that, under the tested conditions, the coating did not significantly change the overall extent of protein removal/adsorption compared with Ti, suggesting comparable overall nonspecific protein-interaction behaviour.

### 3.3 ROS production and phagocytic activity in macrophages on commensal microflora-coated implants

**Fig. 3:**
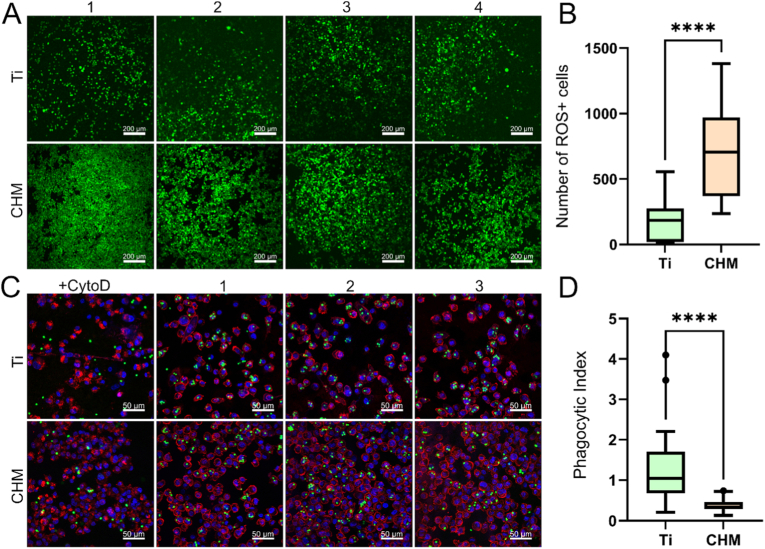
ROS production and phagocytic activity of macrophages on CHMs. (A) Representative images of ROS production in macrophages on Ti and CHM surfaces. (B) Quantitative analysis of ROS generation in macrophages. (C) Representative images of phagocytosis and (D) quantitative phagocytic index of macrophages on Ti and CHM surfaces. All experiments were performed with three biological replicates (N = 3) and three technical replicates per group (n = 3). ROS analysis was conducted at 10× magnification with a voxel size of 1.13 μm (x) × 1.13 μm (y) × 5 μm (z), whereas the phagocytosis assay was imaged at 40× magnification with a voxel size of 0.283 μm (x) × 0.283 μm (y) × 5 μm (z).

Macrophage functional activation on Ti and CHMs was assessed by intracellular reactive oxygen species (ROS) production and phagocytic capacity. Macrophages cultured on Ti displayed low basal ROS signal, whereas cells on CHMs exhibited a clearly increased ROS-positive population, indicating enhanced oxidative burst activity on the coated surfaces (Fig. 3A and B). Phagocytosis assays demonstrated controlled phagocytic activity in macrophages on CHMs, as evidenced by a significant reduction in both signal intensity and the proportion of phagocytically active cells (Fig. 3C and D). Together, these results show that CHM surfaces promote macrophage antimicrobial effector functions, characterized by elevated ROS generation and augmented phagocytosis relative to Ti. Immunostaining revealed that macrophages seeded on Ti and CHMs were positive for surface markers of both M1 and M2 phenotypes (Fig. S5).

### 3.4 Commensal microflora coatings prevent *P. gingivalis* biofilm formation through biofilm-repellent activity

**Fig. 4:**
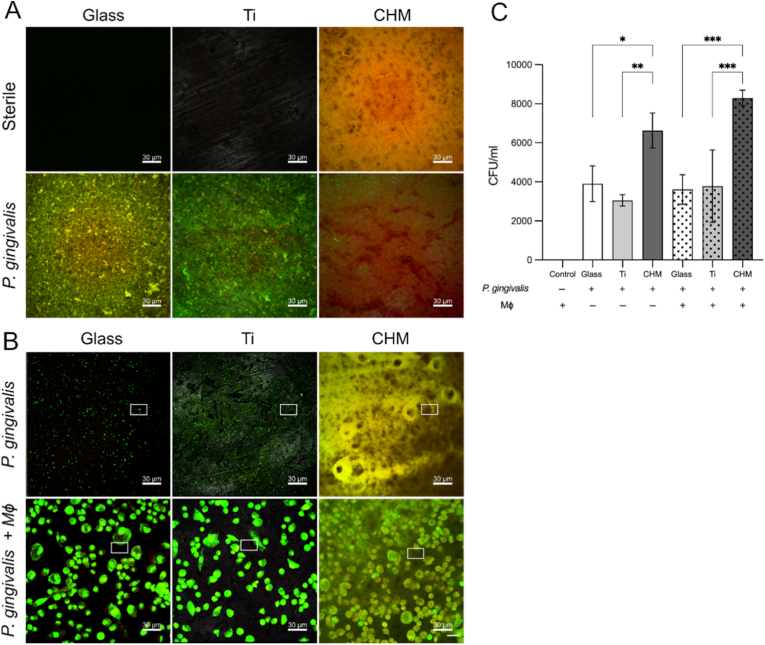
Commensal microflora-coated Ti surfaces repel *P. gingivalis* biofilm formation. (A) CLSM images of glass, Ti, and CHM under sterile conditions and with *P. gingivalis* biofilm (green) after 24 h. (B) CLSM images of planktonic *P. gingivalis* (green) on glass, Ti, and CHMs after 24 h, in monoculture or co-culture with macrophages. Marked regions indicate adherent bacteria. Scale bar: 30 μm. (C) Viable *P. gingivalis* quantified as CFU/mL from culture supernatants after 24 h. Coated implants showed significantly higher CFU counts in supernatants, both with and without macrophages, compared to uncoated controls (∗p ≤ 0.05, ∗∗p ≤ 0.01, ∗∗∗p ≤ 0.001). Representative CFU plates are shown in Supplementary Data. Experiments were performed in three independent biological replicates (N = 3), with three technical replicates (n = 3) per condition. Imaging was conducted at 40× magnification with a voxel size of 0.379 μm × 0.379 μm × 0.999 μm in the x, y, and z dimensions, respectively.

Commensal microflora-coated Ti discs were subjected to live/dead staining to confirm that the coated bacteria were non-viable and posed no risk of sepsis. CLSM imaging showed that the coated surfaces exhibited predominantly red fluorescence due to propidium iodide uptake, confirming that the coating was largely covered by PI-positive (non-viable) bacteria (Fig. 4A–CHM). To evaluate biofilm repellent activities, the biofilm-forming pathogen *P. gingivalis* was incubated on coated and uncoated surfaces for 24 h. CLSM imaging demonstrated abundant viable biofilm on glass coverslips and Ti, whereas coated surfaces predominantly exhibited dead staining with no detectable live *P. gingivalis* (Fig. 4A–CHM).

Since bacterial and host cell adhesion is governed by the concept of the "race for the surface" [43], an additional experiment was performed. *P. gingivalis* was cultured either alone or co-cultured with macrophages on implant surfaces at a multiplicity of infection (MOI) of 1:1 for 24 h. When cultured alone, *P. gingivalis* failed to form biofilms on coated surfaces (Fig. 4B and CHM). In co-cultures, macrophages adhered strongly to coated surfaces, while only few *P. gingivalis* cells were detected (Fig. 4B and CHM, lower side). To quantitatively validate these findings, supernatants from bacterial cultures were analysed for colony-forming units (CFU/mL) (Fig. S6). Interestingly, viable *P. gingivalis* CFUs were significantly higher in the supernatants surrounding coated surfaces compared to glass and titanium (Fig. 4C–CHM). A similar trend was observed in co-culture conditions with macrophages, indicating that CHM coatings reduced bacterial adhesion by repelling *P. gingivalis* into the culture medium (Fig. 4C–CHM). Collectively, these results demonstrate that commensal microflora coatings prevent *P. gingivalis* biofilm formation by providing strong biofilm-repellent activity while promoting host cell adhesion.

### 3.5 Commensal microflora coatings promote osteoimmunomodulatory crosstalk between macrophages and stem cells

**Fig. 5:**
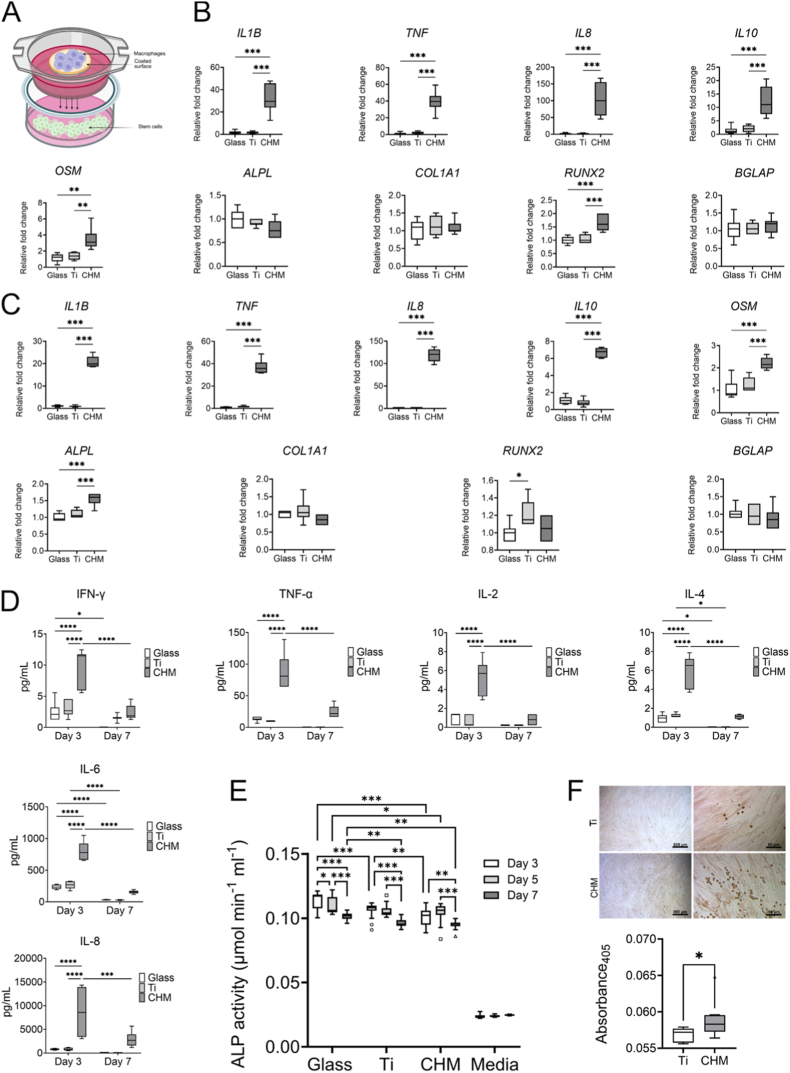
Commensal microflora-coated titanium surfaces induce osteoimmunomodulatory responses in PDLSCs. (A) Schematic representation of the co-culture experimental setup with macrophages and PDLSCs. (B) relative mRNA expression levels of *IL-1β*, *TNF-α*, *IL-8*, *IL-10*, and *OSM* in macrophages, and *ALP*, *COL1*, *RUNX2*, and *BGLAP* in PDLSCs after 3 and 7 days (C) respectively of co-culture on glass, titanium (Ti), CHMs. (D) Multiple cytokine analysis of 2D culture supernatants collected on day 3 and day 7, showing concentrations of IFN-γ, TNF-α, IL-2, IL-4, IL-6, and IL-8. Data are from three independent experiments, each performed with two technical replicates. (E) Alkaline phosphatase (ALP) activity in co-culture media collected after 3, 5, and 7 days. (F) Alizarin red staining of PDLSCs with quantitative analysis. Data are presented as mean ± SD. Statistical significance: ∗p ≤ 0.05, ∗∗p ≤ 0.01, ∗∗∗p ≤ 0.001. Abbreviations: interleukin-1 beta (IL-1β); tumor necrosis factor-alpha (TNF-α); interleukin-8 (IL-8); interleukin-10 (IL-10); oncostatin M (OSM); alkaline phosphatase (ALPL); collagen type I alpha 1 chain (COL1A1); runt-related transcription factor 2 (RUNX2); bone gamma-carboxyglutamate protein/osteocalcin (BGLAP); interferon-gamma (IFN-γ).

Immune cells such as macrophages interact with stem cells to regulate bone healing and regeneration through osteoimmunomodulatory mechanisms [44]. To investigate whether commensal microflora-coated surfaces promote these activities, macrophages and PDLSCs were co-cultured (2D) setting (Fig. 5A). mRNA expression profiles of macrophages were analysed after 3 and 7 days of co-culture. Expression of *IL-1β* and *TNF-α* (M1 markers), as well as *IL-8* and *IL-10* (M2 markers), was significantly upregulated on coated surfaces compared to glass and Ti controls (Fig. 5B). Importantly, macrophages on coated surfaces also showed elevated expression of the osteoinductive cytokine *OSM* (Fig. 5B). Gene expression in PDLSCs was assessed under the same conditions. Co-culture with macrophages seeded on coated surfaces led to significant upregulation of the osteogenic transcription factor *RUNX2* after 3 days compared to controls (Fig. 5B). After 7 days, *RUNX2* expression decreased, while *ALPL* expression significantly increased in *PDLSCs* adjacent to coated surfaces (Fig. 5C). Additionally, mRNA expression of *IL-1β*, *TNF-α*, *IL-8*, *IL-10*, and *OSM* in macrophages remained elevated after 7 days (Fig. 5C). On day 14, PDLSCs cultured with CHMs exhibited a slight decrease in the expression of *ALPL*, *COL1*, *RUNX2*, and *BGLAP* (Fig. S7).

Multiple cytokine profiling of supernatants from the 2D model showed a pronounced, time-dependent response to commensal microflora–coated implants. Overall cytokine levels ‒ encompassing both pro- and anti-inflammatory mediators ‒ peaked at day 3 in the presence of CHMs and declined significantly by day 7, indicating an early, transient immunoregulatory effect. IFN-γ was significantly higher in CHM cultures than on glass or Ti at day 3 and decreased significantly by day 7 (Fig. 5D). A similar pattern was observed for TNF-α, which was maximal at day 3 and significantly reduced at day 7 (Fig. 5D), as well as IL-2, which also declined significantly over time (Fig. 5D). Among anti-inflammatory, IL-4 was significantly elevated in CHM cultures relative to controls at day 3 and subsequently decreased by day 7 (Fig. 5D). In addition, the pro-inflammatory chemokine/cytokines IL-8 and IL-6 were significantly increased at day 3 in CHM conditions and were significantly lower at day 7 (Fig. 5D). IL-10 remained below the assay detection limit at both time points. These data demonstrate that CHMs elicit an early but self-limiting cytokine response, consistent with transient immune activation followed by resolution. To further evaluate osteogenic activity, alkaline phosphatase (ALP) activity in co-culture supernatants was measured on days 3, 5, and 7. Notably, overall ALP activity in media from coated surfaces was significantly lower than that from glass and Ti controls (Fig. 5E). Alizarin red staining of PDLSCs following co-culture with macrophages seeded on CHMs revealed stronger mineralization after 14 days of incubation than in the Ti (Fig. 5F). Collectively, these findings demonstrate that commensal microflora-coated surfaces facilitate immune–stem cells crosstalk by enhancing macrophage activation and stimulating osteogenic differentiation of PDLSCs, thereby exerting a pro-regenerative, osteoimmunomodulatory effect.

### 3.6 Commensal microflora-coated titanium implants maintain biocompatibility and epithelial integrity in 3D human peri-implant tissue models

**Fig. 6:**
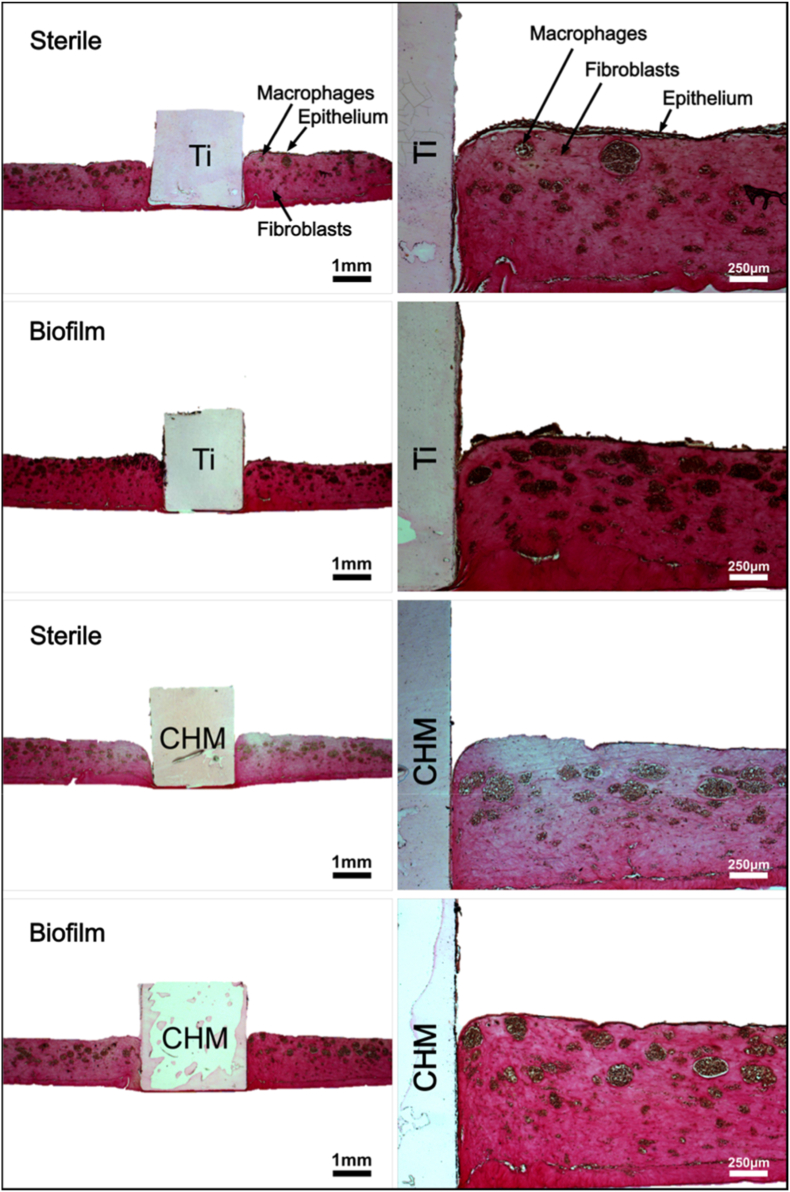
Commensal microflora-coated titanium implants are compatible with a 3D human peri-implant tissue model. Representative sections of 3D tissues formed around Ti and CHMs under sterile conditions or after 24 h exposure to multi-species oral biofilms. Tissue architecture was assessed by Elastica van Gieson staining, highlighting epithelial and connective tissue organization with embedded fibroblasts and macrophages. Experiments were performed in three independent replicates, each with three technical repeats. Scale bars: 1 mm (left), 250 μm (right).

To evaluate the biocompatibility of commensal microflora-coated surfaces, we employed an organotypic 3D INTER_b_ACT model that mimics peri-implant soft tissue [35]. The model consisted of human gingival fibroblasts and THP-1 monocyte-derived macrophages embedded in a gel matrix surrounding titanium implants, overlaid by a stratified epithelium. Titanium cylinders (3 mm in diameter, 2 mm in height) were coated with commensal microflora. SEM revealed a dense and homogeneous microbial layer, while energy-dispersive X-ray spectroscopy confirmed the presence of carbon (Fig. S8). Both Ti and CHM cylinders were then incorporated into the 3D tissue model. Human gingival fibroblasts and macrophages were embedded in the gel matrix, and on day 5 a cylindrical punch was created in the centre to insert either coated or uncoated implants. On day 8, oral epithelial cells were seeded on top of the constructs, which were subsequently cultured at the air–liquid interface for 24 days to allow epithelial stratification and tissue maturation (Fig. S9). Histological analysis using van Gieson staining demonstrated that sterile 3D tissues with Ti developed an intact multi-layered epithelium adjacent to the implant, with fibroblasts distributed throughout the tissue and macrophages forming clusters (Fig. 6, sterile Ti). In biofilm-exposed tissues, the epithelium remained intact, but fibroblasts and macrophages as foci were evident (Fig. 6, biofilm Ti). Similarly, sterile 3D tissues formed around CHMs exhibited an intact epithelium adjacent to the surface, with fibroblasts and macrophages distributed comparably to uncoated controls (Fig. 6, sterile CHM). After biofilm co-culture, CHMs still supported intact epithelial and underlying stromal organization (Fig. 6, biofilm CHM). Live/dead staining further confirmed the viability of cells in both sterile and biofilm-exposed tissues, with green fluorescence indicating living cells (Fig. S10). Fibroblasts adhered to both Ti and CHM implants, and the commensal coating remained visible after co-culture (Fig. S11). Interestingly, macrophages in tissues surrounding both Ti and CHM implants migrated more prominently toward the epithelial layer after biofilm exposure, suggesting enhanced immune activation (Fig. S12). This effect may be partially attributed to the commensal microflora coating, which could prime macrophage responsiveness. Collectively, these results demonstrate that CHMs are biocompatible with human 3D peri-implant soft tissue models under both sterile and biofilm-challenged conditions, while maintaining epithelial integrity and supporting immune cell activity.

### 3.7 Commensal microflora coatings in a 3D human peri-implant tissue model reduce biomass of multi-species biofilms

**Fig. 7:**
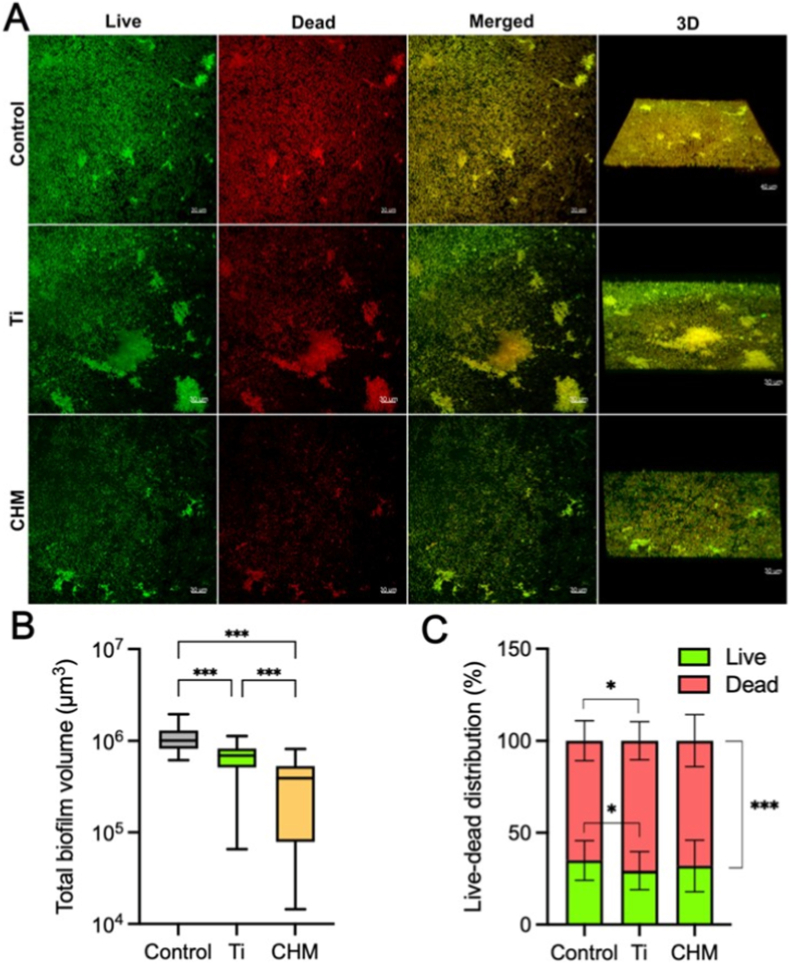
Commensal microflora-coated implants reduce biofilm biomass in 3D co-culture. (A) CLSM images of live (green) and dead (red) stained biofilms grown on glass coverslips under three conditions: (i) control (biofilm without 3D tissue), (ii) co-culture with 3D tissues containing Ti implants, and (iii) co-culture with 3D tissues containing CHM implants. Biofilm morphology is shown in 2D (merged) and 3D views. (B) Quantification of total biofilm volume. (C) Live/dead ratios of biofilms under the indicated conditions. Data represent three independent experiments with three technical replicates each. Statistical significance: ∗p ≤ 0.05, ∗∗p ≤ 0.01, ∗∗∗p ≤ 0.001. CLSM imaging was performed at 40× magnification with a voxel size of 0.379 μm × 0.379 μm × 0.999 μm in the x, y, and z dimensions, respectively.

Following co-cultivation with 3D peri-implant tissues, biofilm morphology was assessed by live/dead staining and visualized using CLSM. Dense biofilms containing both viable and non-viable bacteria were observed on Ti surfaces, comparable to control biofilms formed without tissue co-culture (Fig. 7A, control and Ti). In contrast, 3D tissues with CHMs exhibited markedly reduced biofilm formation. Biofilms on these surfaces appeared thinner, less compact, and more dispersed, indicating a shift toward planktonic growth rather than stable surface-associated communities. Quantitative analysis confirmed a statistically significant reduction in total biofilm volume on CHMs compared to Ti and control surfaces (Fig. 7B). Importantly, live/dead staining showed no substantial differences in bacterial viability between groups, suggesting that the reduced biofilm volume was not due to direct bactericidal activity but rather to the inhibition of bacterial biofilm formation. These findings demonstrate that commensal microflora coatings exert potent biofilm-repellent effects in the presence of host tissues. By showing anti-biofilm activities and potentially enhancing host immune responses within the 3D tissue model, such coatings represent a promising preventive strategy for reducing peri-implant biofilm formation and improving implant outcomes.

### 3.8 Commensal microflora-coated implants orchestrate dynamic macrophage polarization to balance antimicrobial defence and tissue homeostasis

**Fig. 8:**
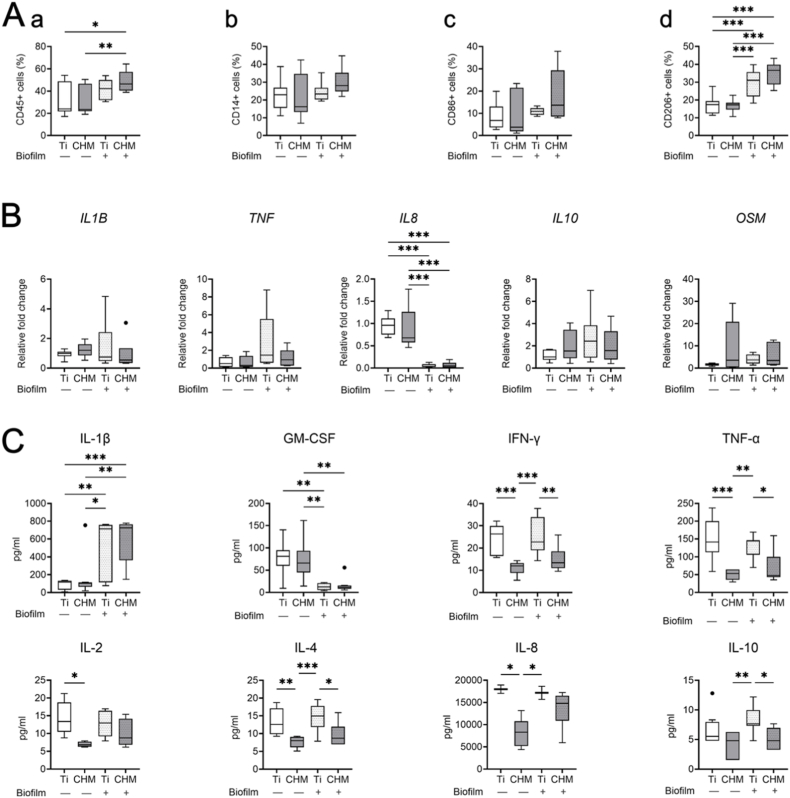
Commensal microflora-coated implants modulate macrophage polarization in 3D tissues. (A) Flow cytometry of macrophages showing CD45^+^ (a), CD14^+^ (M0, b), CD86^+^ (M1, c), and CD206^+^ (M2, d) populations, and (B) mRNA expression of *IL-1β*, *TNF-α*, *IL-8*, *IL-10*, and *OSM* in 3D tissues containing Ti or CHMs, under sterile (−) or biofilm-challenged (+) conditions. (C) Cytokine expression levels of IL-1β, GM-CSF, IFN-γ, TNF-α, IL-2, IL-4, IL-8, and IL-10 in culture supernatants under sterile (−) and biofilm-exposed (+) conditions. Data represent three independent experiments performed at different time points with three technical replicates for each experiment.

The reduction in biofilm volume observed in 3D tissues with CHM implants suggested that the coating influenced macrophage polarization toward either pro-inflammatory (M1) or anti-inflammatory (M2) phenotypes. To investigate this, flow cytometry analysis of macrophages was performed after co-cultivation with 3D tissues under sterile and biofilm-exposed conditions. Analysis of macrophages in the M0 (non-polarized) state revealed a significant increase in CD45^+^ cells in 3D tissues with coated implants exposed to biofilms (Fig. 8Aa), along with a moderate increase in CD14 expression under both sterile and biofilm conditions (Fig. 8Ab). Assessment of M1 polarization showed a marked increase in CD86^+^ macrophages under both sterile and biofilm-exposed conditions in tissues with CHM implants (Fig. 8Ac), indicating enhanced pro-inflammatory activation in response to pathogenic biofilms. M2 polarization, assessed by CD206 expression, showed comparable levels between CHM and Ti implants under sterile conditions. However, upon biofilm exposure, a significant increase in CD206^+^ macrophages was observed in both groups, with a notably higher proportion in tissues surrounding coated implants compared to titanium controls (Fig. 8Ad). Gene expression analysis further supported these observations. mRNA levels of *IL-1β* and *TNF-α* were comparable between CHM and Ti implants (Fig. 8B, *IL-1β* and *TNF-α*). *IL-8* expression significantly decreased upon biofilm exposure (Fig. 8B, *IL-8*), while *IL-10* levels remained unchanged between groups (Fig. 8B, *IL-10*). Importantly, expression of the osteoinductive factor *OSM* was substantially higher in 3D tissues with CHMs under both sterile and biofilm-exposed conditions (Fig. 8B–OSM). Together, these findings indicate that commensal microflora-coated implants dynamically modulate macrophage behaviour in the presence of biofilms. The coating promotes an initial M1-driven pro-inflammatory response to counteract bacterial challenge, followed by a shift toward the M2 phenotype to restore tissue homeostasis. This dual-phase immunomodulatory effect underscores the therapeutic potential of commensal-coated implants in supporting both antimicrobial defence and tissue regeneration within peri-implant environments.

Multiple cytokine profiling of supernatants from the 3D tissue model, assessed under sterile conditions and following exposure to multispecies biofilms, revealed an overall attenuation of inflammatory signaling in the presence of commensal microflora–coated implants. Under sterile conditions, IL-1β expression was comparable between Ti and CHMs and increased significantly following co-cultivation with multispecies biofilms. However, IL-1β expression did not differ significantly between Ti and CHM groups (Fig. 8C, IL-1β). GM-CSF levels were comparable between CHM- and Ti-integrated tissues under sterile conditions and decreased upon co-culture with multispecies biofilms in both groups (Fig. 8C, GM-CSF). Notably, IFN-γ was significantly lower in CHM tissues than in Ti controls under sterile conditions; although IFN-γ increased modestly after biofilm challenge, it remained lower in the CHM group relative to Ti (Fig. 8, IFN-γ). Similarly, TNF-α was significantly reduced in CHM tissues compared with Ti under both sterile and biofilm-exposed conditions (Fig. 8C, TNF-α), and IL-2 followed the same trend. Among cytokines commonly associated with immune regulation, IL-4 and IL-10 were also significantly lower in CHM tissues than in Ti controls under both conditions (Fig. 8C, IL-4 and IL-10). The neutrophil chemoattractant IL-8 was likewise decreased in the CHM group under sterile and biofilm-exposed settings (Fig. 8C, IL-8). IL-6 measurements exceeded the assay's quantitative range in a subset of samples; nevertheless, where quantifiable, IL-6 levels were also lower in CHM tissues (Fig. S13). Overall, these data indicate that CHMs do not provoke an exaggerated cytokine response and instead are associated with globally reduced cytokine production compared with Ti, both at baseline and following multispecies biofilm exposure.

## 4 Discussion

Commensal and probiotic bacteria suppress pathogens through niche competition, antimicrobial metabolite production, and immune modulation, making restoration of a healthy microflora a promising strategy for preventing infection and preserving peri-implant tissue health [45]. In the oral cavity, commensal microfloras contribute to colonization resistance against pathogens, maintain microbiome stability, and support immune balance, thereby promoting peri-implant homeostasis [26,46]. Oral streptococci, as pioneer colonizers, are critical in stabilizing microbial communities and restricting pathogenic overgrowth [32]. Based on this rationale, we developed Commensal Hybrid Materials (CHMs) by immobilizing multilayers of *S. oralis* onto titanium implants using a heat-anchoring process [33]. In our previous studies, CHMs inhibited single- and multi-species biofilm formation by preventing early bacterial adhesion ‒ the critical first step in biofilm establishment ‒ without inducing detectable changes in gene expression, thereby minimizing the risk of promoting antibiotic resistance [34,47]. Moreover, CHMs generated from other oral commensals, such as *Streptococcus salivarius*, reduced pathogenic biofilm adhesion but yielded coating layers that were mechanically and physicochemically unstable beyond 48 h in culture medium. In contrast, *S. oralis* formed a more durable anti-adhesive coating, highlighting the species dependence of coating stability.

In the present study, we investigated the antibiofilm, immunomodulatory, and osteoimmunomodulatory effects of CHMs using oral multispecies biofilms, human cell lines, and an immunocompetent 3D peri-implant tissue model [35]. This model captures the triangular interactions among the implant surface, peri-implant tissue containing immune cells, and multispecies biofilms, thereby providing a clinically relevant platform for studying host–pathogen dynamics in the presence of CHMs. Commensal *S. oralis* was immobilized as multi-layered coatings on titanium through a simple heat-treatment process to minimize the risk of sepsis associated with viable bacteria while ensuring stable surface modification. SEM revealed a dense, adherent, multi-layered coating in which the microorganisms retained their morphology, suggesting preservation of structural integrity and surface functionality. These features indicate that the coating may be sufficiently robust to withstand mechanical stress during implantation while providing durable surface protection. Notably, the coating was rich in carbon, a property associated with improved biocompatibility, mechanical resilience, and long-term stability, consistent with the reported advantages of carbon-based coatings [48,49]. Spectroscopic (FTIR/Raman), surface-chemical (XPS) analyses, and water contact angle measurements indicated that the near-hydrophobic *S. oralis* coating generated a localized biomolecular overlayer on titanium, characterized by protein (amide I/II/III), lipid (C–H), and phosphate/phosphoryl (P–O/PO_2_^−^; P 2p/P 2s) signatures, consistent with cell-envelope and extracellular constituents. This composition aligned with a paraprobiotic design in which key structural motifs are retained after heat inactivation, and it introduced polar, nitrogen- and phosphoryl-rich chemistry that is expected to enhance interfacial hydration‒an established mechanism for reducing nonspecific adsorption and limiting early biofilm initiation [50]. In parallel, these chemical hallmarks supported the likely retention of Gram-positive MAMPs (notably lipoteichoic/teichoic acids, peptidoglycan-associated lipoproteins, and nucleic acids), providing a plausible basis for multimodal innate immune modulation through convergent pattern-recognition receptor pathways [51,52].

Functionally, CHMs reproduced our previous findings and repelled *P. gingivalis* adhesion and biofilm formation while simultaneously supporting macrophage adhesion and growth [34]. Thus, while repelling *P. gingivalis* adhesion, CHMs promote macrophage adhesion to the coated implant surface, which may further contribute to antibacterial effects and support subsequent phases of tissue healing [53] [54]. Live commensal microorganisms and their derivatives are known to competitively inhibit pathogen colonization by occupying ecological niches and binding sites, thereby establishing a protective barrier [16]. In contrast, our results demonstrate that even when immobilized as non-viable coatings, commensal microorganisms retain the ability to prevent the adhesion of biofilm-forming pathogens. Notably, heat treatment did not promote the release of toxic or proinflammatory metabolites from the coated surfaces, as macrophages cultured in CHM-conditioned media showed no morphological changes indicative of polarization, similar to those cultured in Ti-conditioned media. The commensal coating distribution on the entire surface was confirmed by crystal violet staining. The bioburden assay demonstrated that the coated bacteria cells were non-viable, and static as well as dynamic testing in cell culture media confirmed the stability of the coating.

The biocompatibility of the commensal-coated implants was assessed using tissue specific primary human gingival fibroblasts, oral epithelial cells, and THP-1–derived macrophages. The peri-implant soft-tissue seal, formed by epithelial cells overlaying fibroblasts, constitutes a critical barrier that protects osseointegrated implants against bacterial invasion [55,56]. Accordingly, the adhesion, proliferation, and metabolic activity of these cells are key determinants of the potential of novel coatings to integrate with peri-implant tissues. The commensal coating supported the attachment and growth of all three cell types, thereby confirming its biocompatibility. Notably, macrophages exhibited markedly enhanced metabolic activity on the coated surfaces, suggesting improved immune surveillance during the early healing phase and supporting the proposed infection-protective and osteoimmunomodulatory potential of the coating [18]. A haemolysis assay demonstrated favorable blood compatibility of CHMs. Although the haemolysis associated with CHMs was slightly higher than that of the Ti control, the mean value remained below the 2% threshold, indicating good hemocompatibility [57]. This favorable hemocompatibility may enhance material tolerability and reduce the risk of adverse effects, such as thrombus formation. Macrophages cultured on CHMs exhibited increased ROS generation relative to Ti, consistent with enhanced antimicrobial activation, whereas phagocytic activity was reduced, suggesting a lower requirement for active engulfment rather than diminished immune competence [58]. Together, these findings indicate that CHMs promote a regulated antimicrobial and pro-regenerative macrophage response that may support tissue healing while limiting excessive inflammation and fibrotic foreign body responses.

Osteoimmunomodulatory implant coatings can promote osseointegration by directing the early immune response toward a pro-regenerative macrophage phenotype, thereby limiting excessive inflammation while enhancing the osteogenic microenvironment required for bone formation and long-term implant stability [59]. To further substantiate this osteoimmunomodulatory function, macrophages cultured on the CHMs were subsequently assessed for their ability to regulate PDLSC behavior. By day 3 and 7, macrophages cultured on CHMs exhibited significantly increased mRNA expression of *IL-1β, TNF-α, IL-8,* and *IL-10* compared with those cultured on Ti. Whereas *IL-1β* and *TNF-α* are indicative of M1-like pro-inflammatory activation, *IL-10* is commonly associated with the anti-inflammatory M2 phenotype [60]. This concurrent expression of M1-and M2-associated markers suggests a balanced immune profile capable of both infection control and early tissue repair. In addition, macrophages on the coated surfaces showed elevated expression of oncostatin M (*OSM*), a cytokine that promotes osteogenesis by driving stem cell differentiation toward the osteoblastic lineage through upregulation of *RUNX2*, *ALP*, and *OCN* [61]. These observations were further corroborated in the macrophage–PDLSC co-culture model. Notably, stem cells co-cultured with macrophages on CHMs exhibited significantly increased *RUNX2* expression on day 3 relative to control conditions, suggesting that CHM-primed macrophages create a microenvironment that supports the early osteogenic differentiation of PDLSCs. *RUNX2* is a master osteogenic transcription factor essential for mesenchymal-to-osteoblast differentiation, controlling alkaline phosphatase, osteocalcin, and collagen type I expression [62]. This finding is consistent with previous studies demonstrating that macrophage–stem cells crosstalk, mediated by factors such as Oncostatin M and IL-10, enhances RUNX2-driven osteogenesis in mesenchymal stem cells [63,64].

Despite the observed increase in *RUNX2* mRNA expression, alkaline phosphatase (ALP) activity remained unchanged in stem cells cultured with the coated implants. This indicated that the coating preferentially promoted early osteogenic commitment, whereas downstream maturation-associated markers such as ALP may remain stable at the early time points assessed, consistent with the stage-dependent kinetics of osteogenic differentiation in periodontal ligament cells reported in another study [65]. The multiple cytokine analysis of culture supernatants indicated that CHMs elicited an early, self-limiting release of cytokines (IFN-γ, TNF-α, IL-2, IL-6, IL-8 and IL-4) that peaked at day 3 and declined by day 7, consistent with a transient innate activation followed by resolution. Such kinetics are in line with established concepts in biomaterial immunology, whereby macrophages initially mount an early inflammatory program, including TNF-α-, IL-6-, and IFN-γ-associated signaling, that subsequently contracts as the system equilibrates, particularly when the stimulus is non-replicative and/or rapidly contained at the biomaterial interface [66]. Collectively, these findings suggest that CHMs may sustain an infection-resistant and pro-regenerative microenvironment that supports new bone formation alongside tissue healing.

The 3D human peri-implant tissue model used to evaluate the performance of CHMs incorporates immune cells and therefore provides a highly relevant *in vitro* platform for investigating early host responses to innovative implant coatings [35,67]. The model recapitulates the complex architecture of the peri-implant soft tissue barrier, enabling simultaneous evaluation of epithelial sealing, fibroblast–matrix interactions, and immune cell activity [68,69]. In addition, it allows controlled co-culture with multi-species biofilms, thereby closely reflecting clinically relevant microbial challenges [70]. 3D tissues developed around CHMs remained intact after three weeks of cultivation, with preserved cellular organization: fibroblasts exhibited organized spreading, macrophages retained clustering, and epithelial cells formed cohesive multi-layered structures. This morphology closely resembled that observed around Ti, confirming biocompatibility and the ability of the coating to support peri-implant cell types within a complex microenvironment. Importantly, the epithelial seal adjacent to CHMs was maintained, indicating that the commensal coating stabilized epithelial integrity, promoted mucosal homeostasis, and reduced the risk of barrier breakdown. Upon challenge with a dysbiotic multi-species biofilm containing *P. gingivalis* [71], epithelial disruption was observed in 3D tissues formed around the titanium. Consistent with earlier reports, analogous 3D biofilm co-culture models exposed to pathogenic biofilms have demonstrated marked epithelial damage and dysbiosis [[72], [73], [74], [75]]. By contrast, the protective effect observed for CHMs aligns with the physiological role of commensal microflora in maintaining symbiosis and supporting tissue homeostasis [76]. Consistently, tissues containing CHMs exhibited significantly reduced pathogenic biofilm biomass and volume than tissues around Ti.

Moreover, under dysbiotic conditions, coated surfaces promoted macrophage polarization into both M1 and M2 phenotypes, reflecting a balanced immune profile. M1 macrophages contribute to antimicrobial defense, whereas M2 macrophages promote repair and resolution of inflammation [77]. This dual polarization may be particularly advantageous for maintaining peri-implant tissue health without triggering excessive inflammatory responses. Supporting this interpratation, commensal metabolites such as butyrate are known to enhance M2 activation [78], and oral commensals have been shown to predominantly elicit M2-skewed responses [79]. Importantly, CHMs did not increase the expression of the pro-inflammatory gene *IL-1β, TNF-α* in 3D tissues, while markedly elevating OSM expression, a cytokine that drives osteogenesis through upregulation of *RUNX2, ALP,* and *OCN* [80].

Multiple cytokine analysis of 3D tissue culture supernatants revealed an attenuated cytokine milieu in the presence of CHMs compared to Ti, both under sterile conditions and following multispecies biofilm challenge, including lower levels of IFN-γ, TNF-α, IL-2, and IL-8, as well as IL-6 where quantifiable. This dampened inflammatory profile may reflect the greater physiologic buffering capacity of the 3D microenvironment, including cell–cell and cell–extracellular matrix interactions as well as diffusion constraints, and, importantly, a reduced propensity of CHMs to amplify pro-inflammatory cascades associated with implant-related foreign body responses [81]. Mechanistically, these findings are consistent with the paraprobiotic concept, whereby heat inactivation abolishes microbial replication while preserving envelope-associated molecular patterns capable of engaging innate immune receptors, such as TLR2-mediated sensing of Gram-positive surface ligands, in a manner that favors controlled, homeostatic signaling rather than sustained inflammation [82]. Notably, oral streptococci, including *S. oralis*, are increasingly recognized as immunologically active commensals capable of shaping host responses, supporting the plausibility that a non-viable *S. oralis* layer can modulate macrophage cytokine outputs at the implant interface [83]. Collectively, these findings indicate that commensal coatings not only preserve epithelial architecture but also restrict pathogen overgrowth, attenuate inflammation, and exert immunomodulatory effects.

Although our study demonstrates that CHMs elicit consistent and favorable biological effects, clinical translation will require scalable manufacturing with defined composition, stable surface chemistry, and high batch-to-batch reproducibility. Achieving this will depend on the development of a standardized, quality-controlled fabrication workflow, including bacterial cultivation, validated heat-inactivation conditions, and surface immobilization under tightly controlled physicochemical parameters. Because CHM coatings are bio-derived and contain protein- and phosphate-rich constituents, terminal sterilization may alter surface organization and chemistry, potentially compromising bioactivity. Accordingly, sterilization strategies should be selected and optimized not only on the basis of sterility assurance but also with regard to preservation of coating integrity, surface composition, and biological function.

From a translational perspective, heat inactivation eliminates bacterial replication while retaining the capacity for controlled host immune engagement, thereby offering an important safety advantage for clinical application. Nevertheless, inter-individual differences in immune tone and local inflammatory status may influence host responses to the coating. For this reason, stepwise *in vivo* validation should prioritize both safety and immunological profiling alongside efficacy, including comprehensive biocompatibility assessment with local and systemic endpoints, time-resolved characterization of peri-implant immune cell composition and cytokine signatures to distinguish transient beneficial activation from persistent inflammation, and evaluation across relevant implantation sites and, where appropriate, higher-fidelity peri-implant models.

To translate these *in vitro* findings toward clinical relevance, we plan to undertake a next phase of investigation that integrates mechanistic profiling with *in vivo* validation and infection-challenge studies using multispecies biofilms. In established titanium implantation models, including rod or screw implants placed in long-bone sites, CHMs will be assessed at multiple time points to characterize early tissue responses, including non-invasive longitudinal monitoring of IFN-β signaling in transgenic reporter mice, as well as analysis of inflammatory infiltrates, fibrous encapsulation, and osseointegration outcomes [84]. For dental applications, subsequent jaw implant models, selected according to feasibility and ethical considerations, will enable evaluation of peri-implant mucosal biology and tissue homeostasis in a more clinically relevant setting [85]. Collectively, this stepwise strategy will help determine whether CHM coatings can deliver durable antimicrobial protection while maintaining a pro-regenerative host response conducive to long-term implant integration.

## 5 Conclusion

Our findings demonstrate that commensal hybrid materials transform otherwise inert implant surfaces into stable, biologically active interfaces capable of resisting biofilm formation, supporting host cell integration, and orchestrating balanced immune and osteogenic responses. By combining anti-infective protection with immune modulation and bone-regenerative potential, CHMs represent a translationally relevant strategy to improve implant longevity and reduce the burden of peri-implant disease.

## Ethics approval and consent to participate

Human blood was obtained from a healthy volunteer after informed consent, in accordance with the Declaration of Helsinki and with ethical approval from the Ethics Committee of Hannover Medical School, Germany under internal review number 11824-BO-K-2025. Cell lines used in this study (THP-1, HGFs, OKF6/TERT-2, PDLSCs) were commercially obtained or previously established and did not require additional ethical approval.

## Funding source

Mainly funded by the 10.13039/501100001659Deutsche Forschungsgemeinschaft (DFG, German Research Foundation) – SFB/TRR-298-SIIRI – Project-ID 426335750 and ALLEGRO program offered by Hannover Medical School funded from the Ministry of Science and Culture of Lower Saxony, Germany.

## CRediT authorship contribution statement

**Raunak Lohar:** Formal analysis, Investigation, Methodology, Visualization. **Tayyaba Nawaz:** Methodology. **Timm Landes:** Investigation. **Paula Schaefer-Dreyer:** Methodology. **Philipp-Cornelius Pott:** Methodology, Writing – review & editing. **Michael Pflaum:** Methodology, Writing – review & editing. **Meike Stiesch:** Conceptualization, Formal analysis, Funding acquisition, Investigation, Project administration, Resources, Supervision, Validation, Visualization, Writing – original draft, Writing – review & editing. **Muhammad Imran Rahim:** Conceptualization, Data curation, Formal analysis, Funding acquisition, Investigation, Methodology, Project administration, Supervision, Validation, Visualization, Writing – original draft, Writing – review & editing.

## Declaration of competing interest

The authors declare the following financial interests/personal relationships which may be considered as potential competing interests:

Raunak Lohar, Muhammad Imran Rahim, Meike Stiesch reports financial support was provided by ALLEGRO, DFG, German Research Foundation. Muhammad Imran Rahim, Meike Stiesch has patent #Patent Application No. WO2020234332A1, EP20725724.7A, US20220211906A1 pending to United States. If there are other authors, they declare that they have no known competing financial interests or personal relationships that could have appeared to influence the work reported in this paper.
