# Supplementary material for: Commensal microbiota–coated biohybrid implants induce antibiofilm, osteogenic, and immunomodulatory responses in a human 3D immunocompetent model
Source: Bioact Mater. 2026 Jun 16;65:626–43. doi: 10.1016/j.bioactmat.2026.06.006 (PMC13284439; doi:10.1016/j.bioactmat.2026.06.006)
Supplement: Multimedia component 1 [file mmc1.docx]

#### **Commensal Microbiota–Coated Biohybrid Implants Induce Antibiofilm, Osteogenic, and Immunomodulatory Responses in a Human 3D Immunocompetent Model**

#### Raunak Lohar^1,2^, Tayyaba Nawaz^1,2^, Timm Landes^3^, Paula Schaefer-Dreyer^1,2^, Philipp-Cornelius Pott^1,2^, Michael Pflaum^2,4^, Meike Stiesch*^1,2^, Muhammad Imran Rahim*^1,2^

^1^Department of Prosthetic Dentistry and Biomedical Materials Science, Hannover Medical School, Carl-Neuberg-Str. 1, 30625 Hannover, Germany.

^2^Lower Saxony Centre for Biomedical Engineering, Implant Research and Development (NIFE), Stadtfelddamm 34, 30625 Hannover, Germany.

^3^ HOT – Hannover Centre for Optical Technologies, Leibniz University Hannover, Nienburger Str. 17, 30167 Hannover, Germany.

^4^Clinic for Cardiothoracic, Transplantation and Vascular Surgery, Hannover Medical School, Carl-Neuberg-Str. 1, 30625 Hannover, Germany.

#### *Correspondence authors:

#### Email: Stiesch.Meike@mh-hannover.de

#### E-mail: Rahim.Muhammad@mh-hannover.de

#### Tel.: +49 (0) 511 532 7288


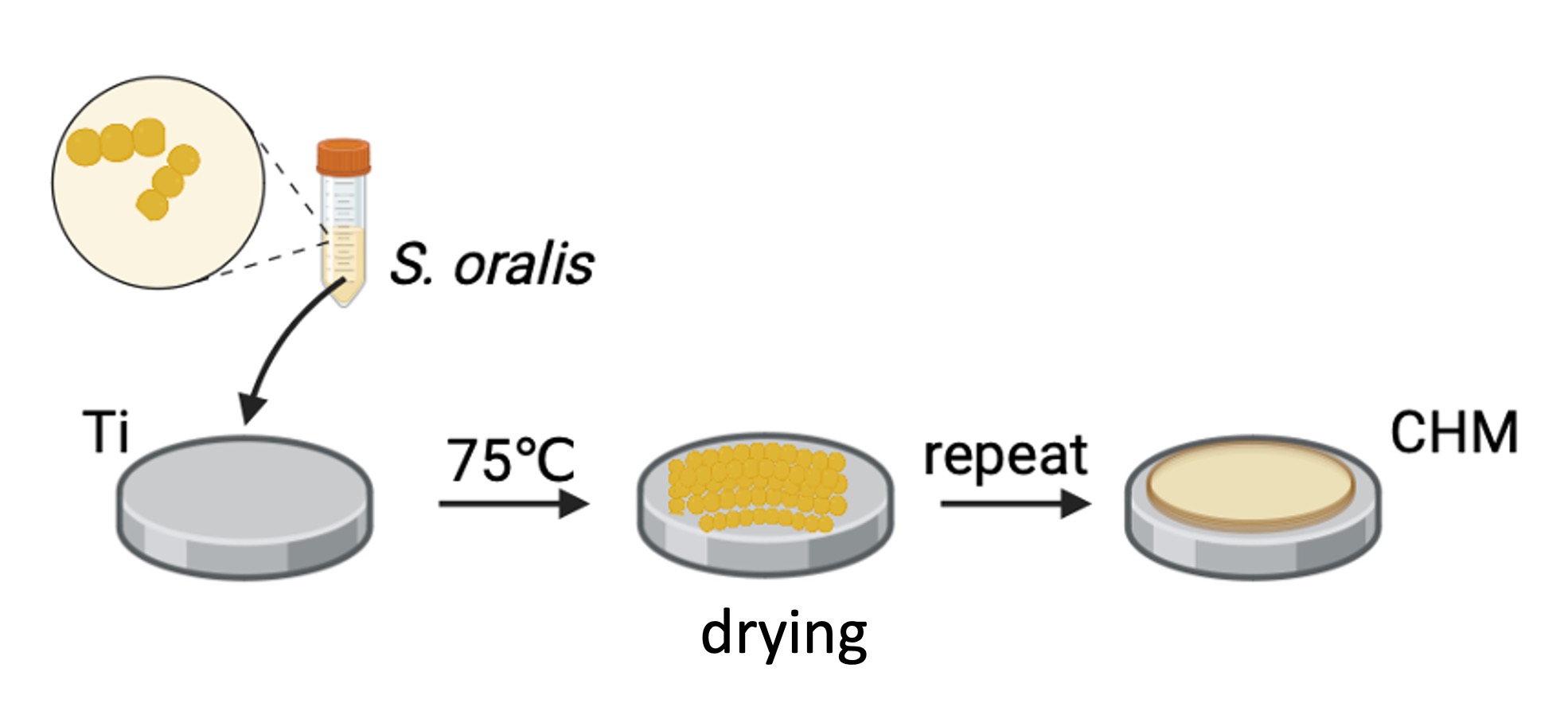


**Fig. S1.** Development of Commensal Hybrid Materials (CHMs). Schematic illustration showing the preparation of commensal coatings: *S. oralis* cells were harvested by centrifugation, washed, and subsequently applied in multiple layers onto titanium surfaces placed on a hot plate at 75 °C, resulting in the formation of stable commensal microflora coatings.


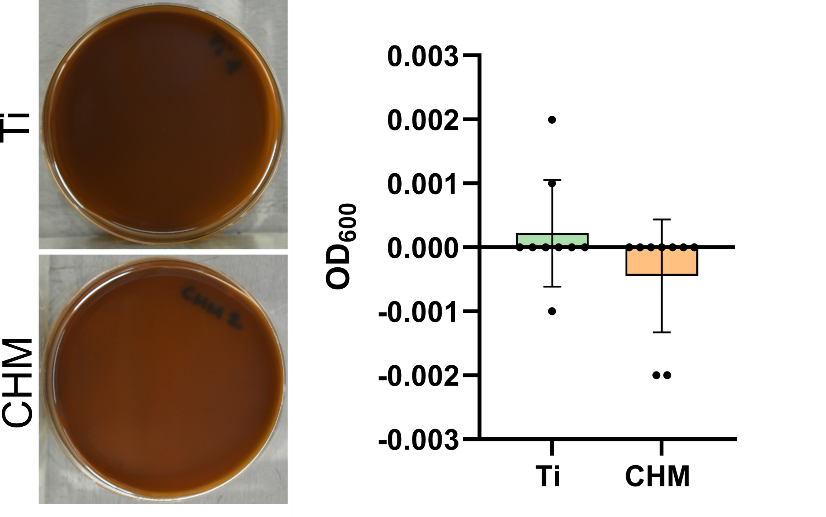


**Fig.S2.** Bioburden assay of CHMs. Colony-forming units (CFU) and optical density at 600 nm (OD_600_) were measured in the surrounding medium of titanium (Ti) and CHM surfaces.


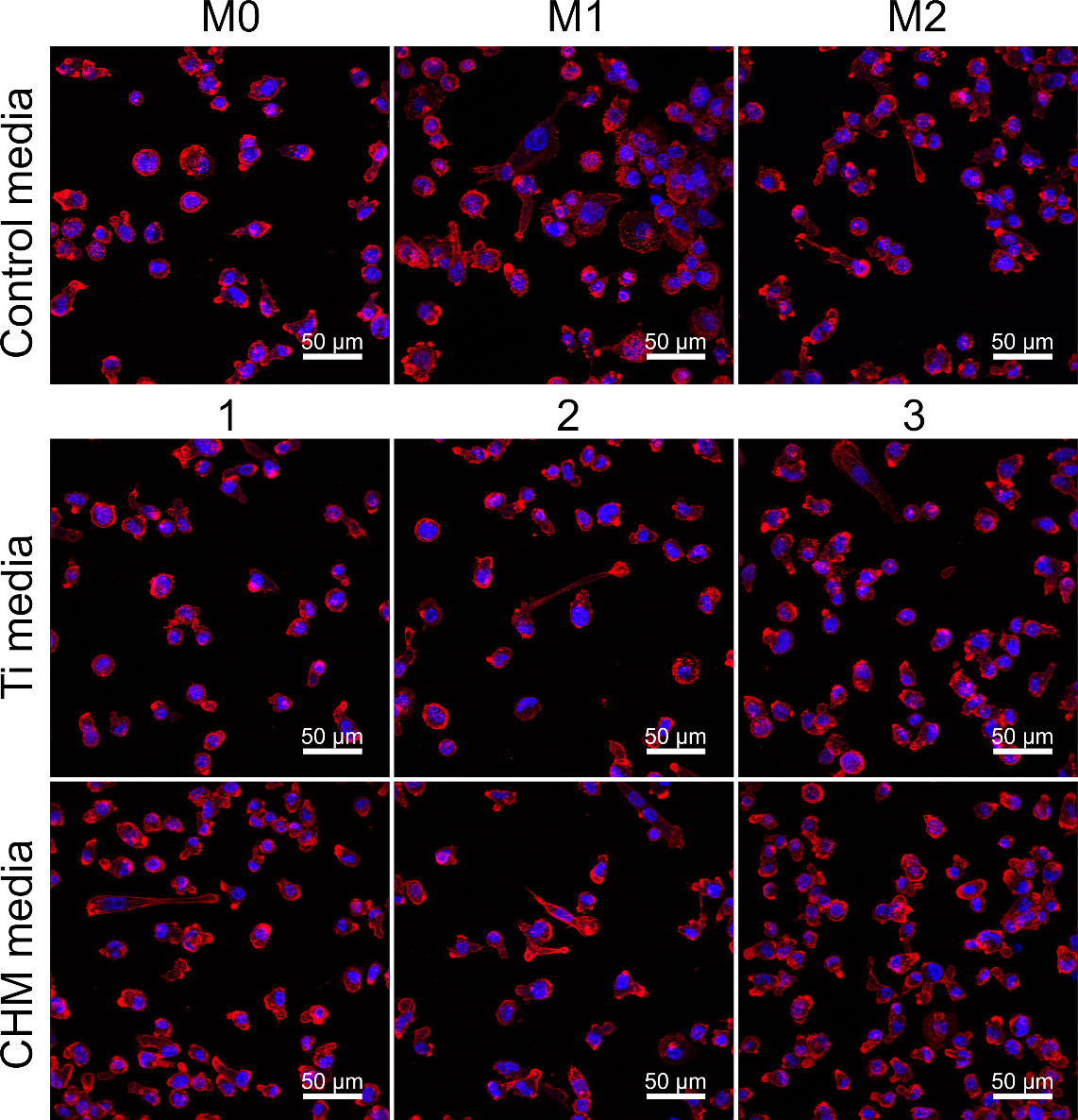


**Fig. S3.** Morphology of macrophages cultured in conditioned media incubated with CHM and titanium surfaces.


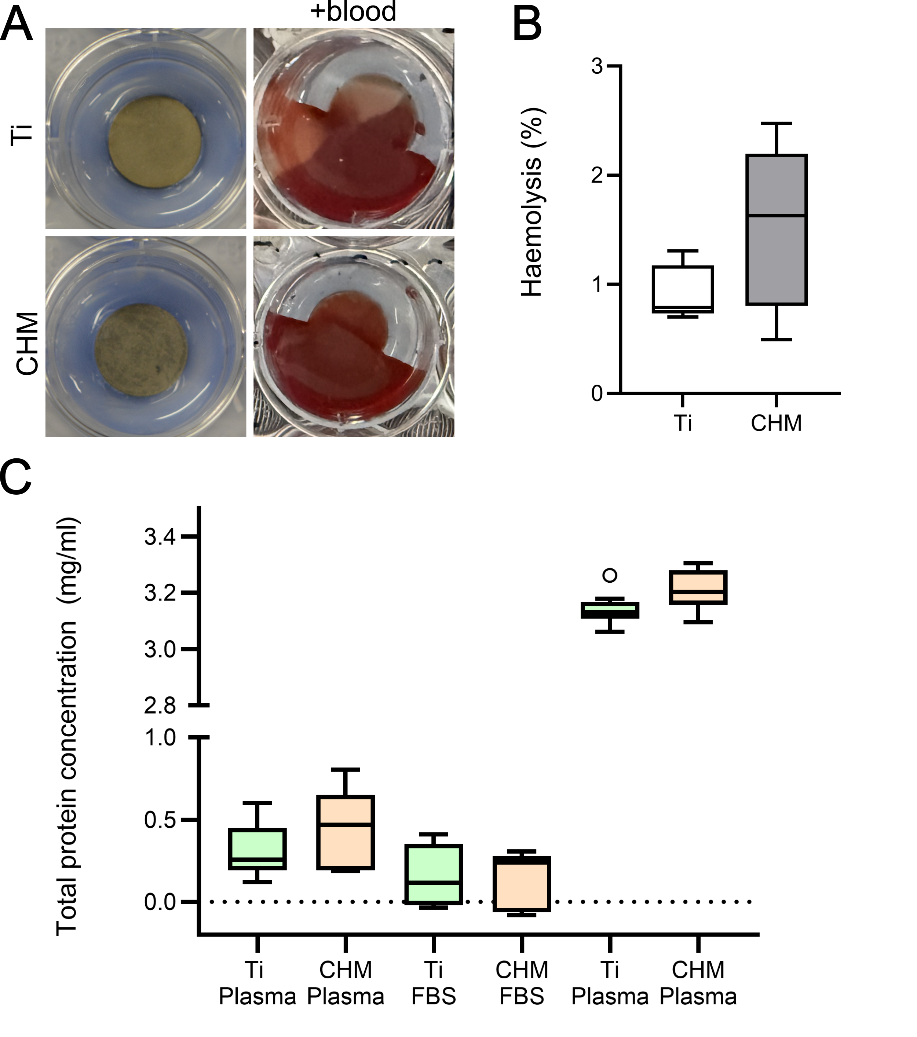


**Fig.S4.** (A) Ti and CHM surface before and after incubation with human blood, (b) percentage haemolysis of Ti and CHMs. (C) Bradford assay showing the adsorption of proteins from human plasma collected before and after 24 h of incubation. Bovine fetal serum (FBS) was also used to assess protein adhesion on Ti and CHMs.


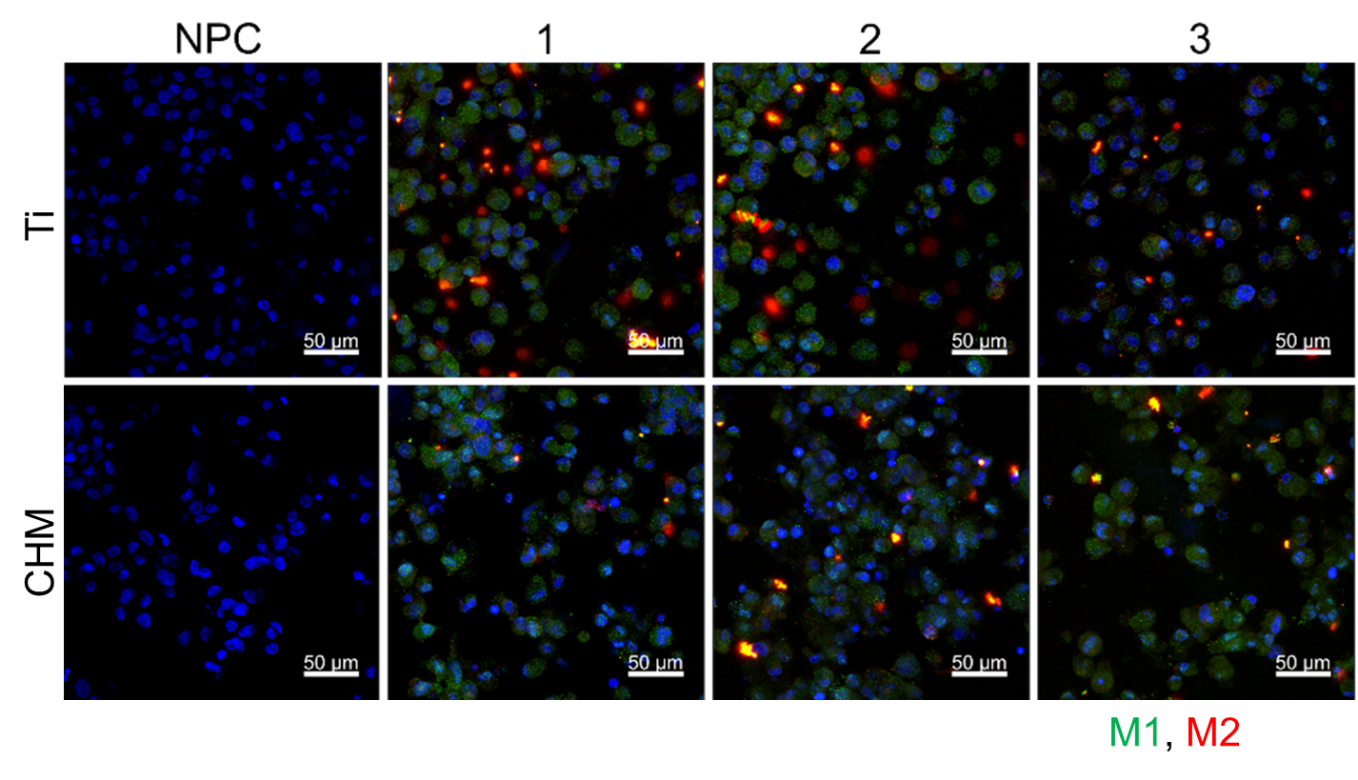


**Fig.S5.** Immunostaining of macrophages on titanium and CHM surfaces, showing M1 markers in green and M2 markers in red.


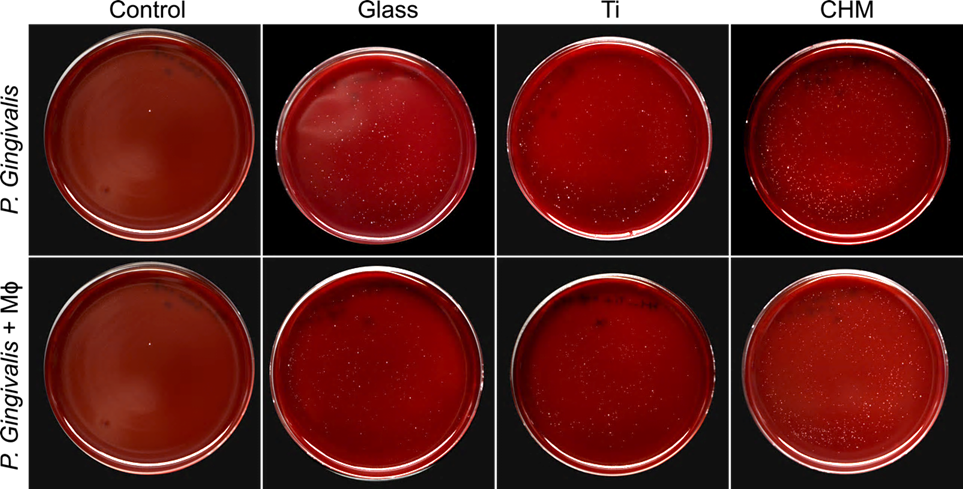


**Fig. S6.** Colony-forming-units of *P. gingivalis* after 5 days due to plate-spreading of supernatants extracted post its 24-hour culture on glass, Ti and CHM surfaces in the presence and absence of macrophages. FAA blood agar used as solid growth media.


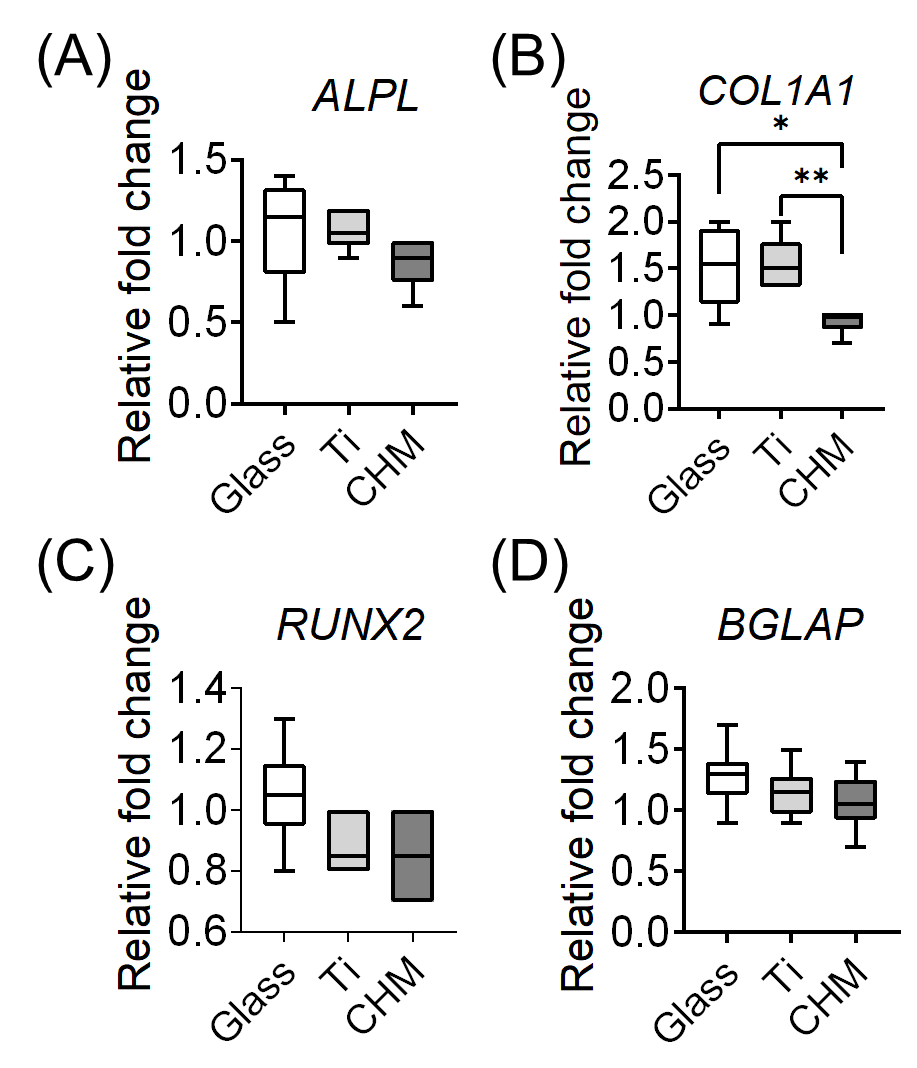


**Fig. S7.** Multiplex cytokine profiling of culture supernatants from the macrophage–stem cell co-culture model after 14 days of culture.


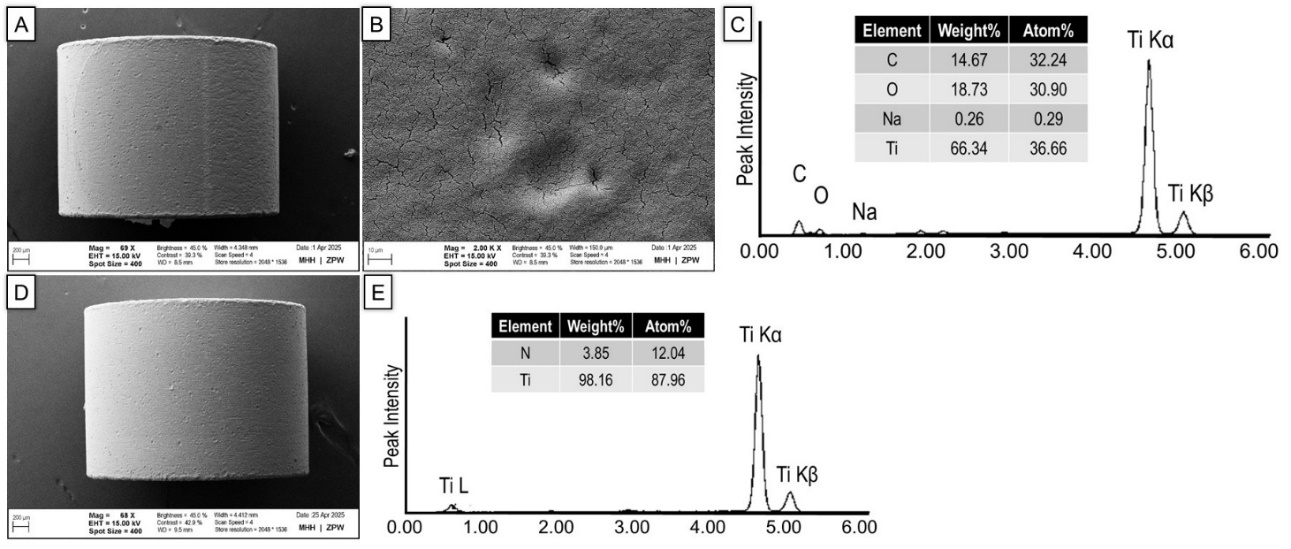


**Fig. S8.** Scanning electron microscopy of CHM (A,B) and EDS analysis (C) SEM of Ti (D) and EDS analysis (E).


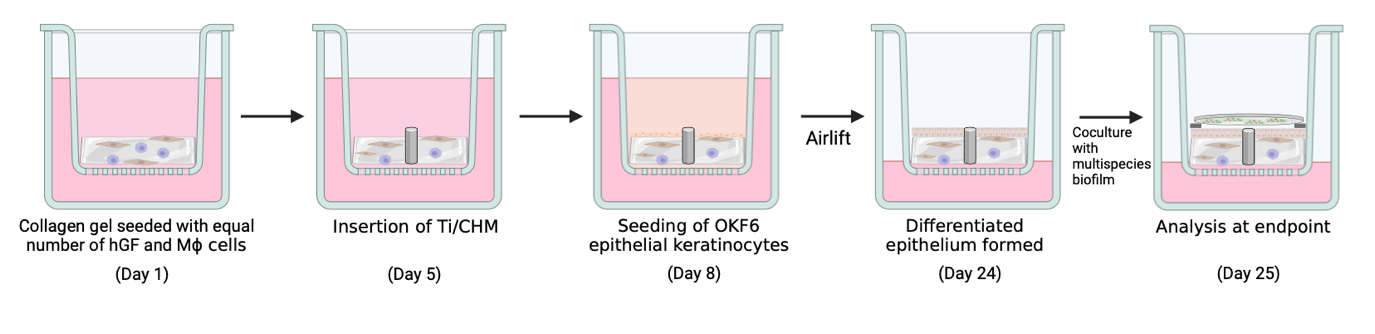


**Fig. S9.** Development of the human cell-line based 3D peri-implant mucosa (INTER_b_ACT) model for the analysis of Ti and CHMs, followed by a 24 h coculture with multispecies bacterial biofilm (*Streptococcus oralis*, *Veillonella dispar*, *Porphyromonas gingivalis* and *Actinomyces naeslundii*).


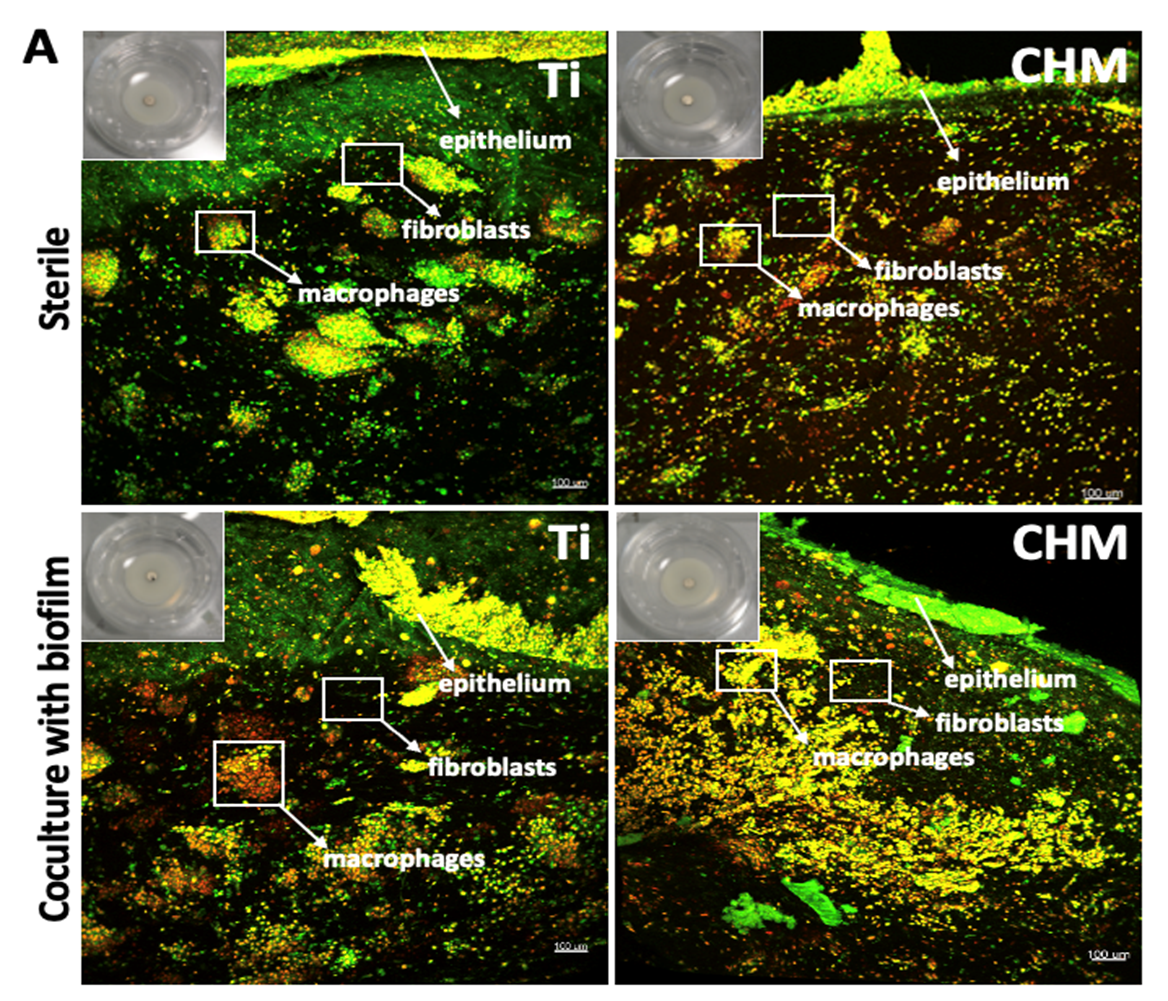


**Fig. S10.** Live-dead staining images of mature peri-implant mucosa tissue showing the top epithelial layer, interspersed fibroblasts and clusters of macrophages (inflammation foci), Inlaid image ‒ top view of mature peri-implant mucosa.


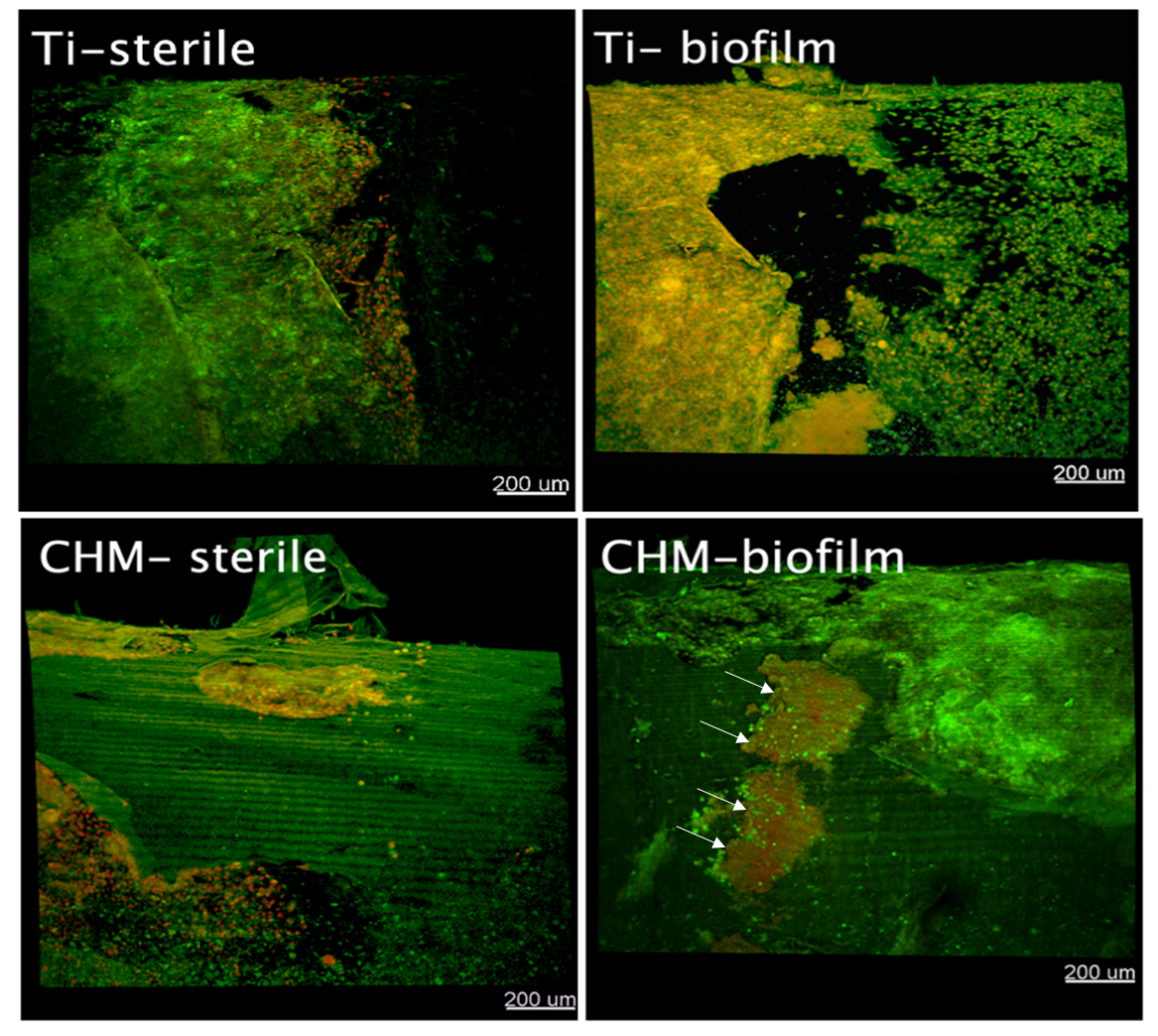


**Fig. S11.** Ti and CHM implants post tissue integration and co-culture showing adhered cells and coating presence.


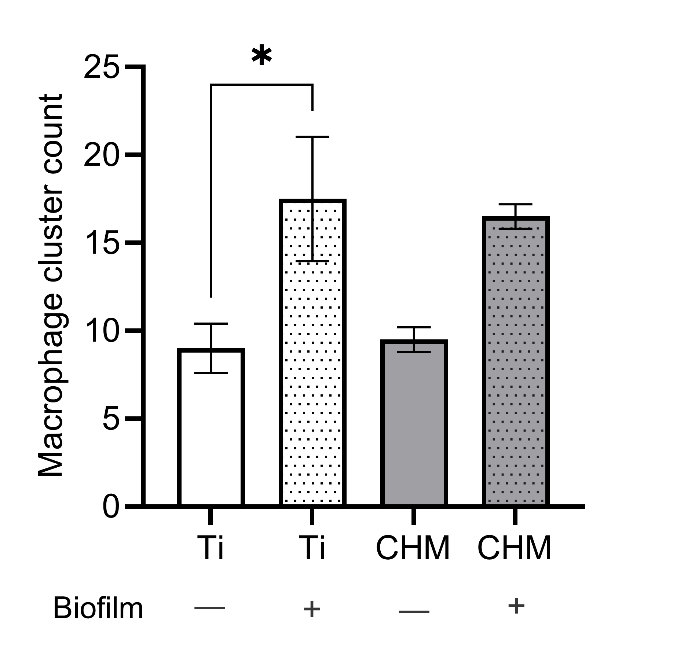


**Fig. S12.** Macrophage cluster count in 3D tissues with Ti and CHM without (-) and with biofilms (+).

**Fig. S13.** IL-6 expression in Ti and CHMs under sterile and biofilm-exposed conditions.

**Supplementary Table 1**: List of primers used for RT-PCR analysis

| Gene name | Gene symbol | TaqMan® gene expression assay ID | NCBI reference sequence |
| --- | --- | --- | --- |
| Glyceraldehyde-3-phosphate dehydrogenase | *GAPDH* | Hs99999905_m1 | NM_001289746.1/ NM_001289746.2 |
| Interleukin 1 beta | *IL 1B* | Hs01555410_m1 | NM_000576.2 |
| Tumor necrosis factor | *TNF* | Hs00174128_m1 | NM_000594.3/ NM_000594.4 |
| C-X-C motif chemokine ligand 8 | *CXCL8 (IL 8)* | Hs00174103_m1 | NM_000584.3 |
| Interleukin 10 | *IL 10* | Hs00961622_m1 | NM_000572.2/ NM_000572.3 |
| Oncostatin M | *OSM* | Hs00171165_m1 | NM_001319108.1/ NM_001319108.2 |
| Alkaline phosphatase | *ALPL* | Hs01029144_m1 | NM_000478.5/ NM_000478.6 |
| Collagen type I alpha 1 | *COL1A1* | Hs00164004_m1 | NM_000088.3/ NM_000088.4 |
| Runt related transcription factor 2 | *RUNX2* | Hs01047973_m1 | NM_001015051.3/ NM_001015051.4 |
| Bone gamma-carboxyglutamate protein | *BGLAP (OCN)* | Hs01587814_g1 | NM_199173.5/ NM_199173.6 |
